# Modeling land use change and forest carbon stock changes in temperate forests in the United States

**DOI:** 10.1186/s13021-021-00183-6

**Published:** 2021-07-03

**Authors:** Lucia A. Fitts, Matthew B. Russell, Grant M. Domke, Joseph K. Knight

**Affiliations:** 1 Department of Forest Resources, University of Minnesota, St. Paul, MN 55108 USA; 2 Northern Research Station, USDA Forest Service, St. Paul, MN 55108 USA

**Keywords:** Ecosystem services, Carbon dynamics, Forest loss drivers, Forest inventory, Remote sensing, USDA Forest Inventory and Analysis (FIA) data

## Abstract

**Background:**

Forests provide the largest terrestrial sink of carbon (C). However, these C stocks are threatened by forest land conversion. Land use change has global impacts and is a critical component when studying C fluxes, but it is not always fully considered in C accounting despite being a major contributor to emissions. An urgent need exists among decision-makers to identify the likelihood of forest conversion to other land uses and factors affecting C loss. To help address this issue, we conducted our research in California, Colorado, Georgia, New York, Texas, and Wisconsin. The objectives were to (1) model the probability of forest conversion and C stocks dynamics using USDA Forest Service Forest Inventory and Analysis (FIA) data and (2) create wall-to-wall maps showing estimates of the risk of areas to convert from forest to non-forest. We used two modeling approaches: a machine learning algorithm (random forest) and generalized mixed-effects models. Explanatory variables for the models included ecological attributes, topography, census data, forest disturbances, and forest conditions. Model predictions and Landsat spectral information were used to produce wall-to-wall probability maps of forest change using Google Earth Engine.

**Results:**

During the study period (2000–2017), 3.4% of the analyzed FIA plots transitioned from forest to mixed or non-forested conditions. Results indicate that the change in land use from forests is more likely with increasing human population and housing growth rates. Furthermore, non-public forests showed a higher probability of forest change compared to public forests. Areas closer to cities and coastal areas showed a higher risk of transition to non-forests. Out of the six states analyzed, Colorado had the highest risk of conversion and the largest amount of aboveground C lost. Natural forest disturbances were not a major predictor of land use change.

**Conclusions:**

Land use change is accelerating globally, causing a large increase in C emissions. Our results will help policy-makers prioritize forest management activities and land use planning by providing a quantitative framework that can enhance forest health and productivity. This work will also inform climate change mitigation strategies by understanding the role that land use change plays in C emissions.

**Supplementary Information:**

The online version contains supplementary material available at 10.1186/s13021-021-00183-6.

## Background

Forests, as part of their ecosystem services, serve as the world’s largest terrestrial sink of carbon (C) by storing it in biomass and soil [1–3]. This C cycles through the ecosystem via biogeochemical processes causing it to move between different pools, (i.e., aboveground and belowground biomass, dead wood, litter, organic soil, and harvested wood products) or back to the atmosphere depending on the ecosystem’s dynamics and disturbances. These processes include photosynthesis, respiration, decomposition, and natural and anthropogenic disturbances [4]. Studies of C stocks in forests are key to informing changes in greenhouse gas emission and removal accounts under climate change scenarios [3] and can be estimated at different scales.

When quantifying C at a large geographic scale, land use and land cover change and disturbance history are essential components to consider [3, 5, 6]. In this matter, land use dynamics [2, 6–8], including land use legacies [7, 9], are major factors affecting terrestrial C fluxes. For example, C accumulation in forests of the eastern United States has been credited to historical changes in land use, especially to forest regrowth after agricultural abandonment [9]. However, when quantifying C at a stand-level scale, land use change is rarely incorporated [10], creating uncertainties in C accounting.

Carbon emissions due to land use change can be quantified by separating the different C fluxes into its individual components. The Intergovernmental Panel on Climate Change (IPCC) provides guidelines for the estimation of national greenhouse gas (GHG) inventories [11], including C, which are consistent with reporting requirements in the United Nations Framework Convention on Climate Change (UNFCCC). These guidelines specifically include operational models to estimate fluxes (emissions and removals) using a process for each C pool. The fluxes are estimated for each land use category and differentiate the categories remaining the same from those that were converted to another land use.

However, much uncertainty exists when quantifying how much land use and land cover change actually contributes to C flux [2, 12], due, in part, to a lack of confidence in separating these fluxes into individual components [12]. In addition, C dioxide emissions or transfers resulting from land use change may be underestimated as some processes (e.g., tree harvesting and land clearing from shifting cultivation) are not considered [9, 12–14] or data is limited. Similarly, there are too few global-scale observational constraints to exclusively estimate anthropogenic land use and land cover C emissions [12]. Different methods have been used to estimate changes in C density caused by land use change. Three of the most common approaches include: inventory-based estimates, satellite-based estimates, and process-based vegetation models [14]. In the US, estimates of GHG emissions and removals are estimated with data from the Forest Inventory and Analysis (FIA) program, which is in charge of conducting the US national forest inventory [4].

One of the common measures used in land use change research is land cover change, though this does not always accurately reflect actual land use change [15]. A crucial difference between these two concepts is that tree cover loss (in case of forest cover) does not always show the activities that actually happen on the ground. Different drivers affect land use and land cover change. On the one hand, global drivers of tree cover loss include deforestation (mostly in Southeast Asia and Latin America), shifting agriculture (Africa and Latin America), large-scale forestry operations (Europe, North America, Russia/China/Southeast Asia, and Australia/Oceania), wildfire (Russia/China/Southeast Asia, Australia/Oceania, and North America), and urbanization (North America) [16]. On the other hand, land use change is caused by both human and climate drivers. Decisions on land use are often based on short-term economic factors and are influenced by globalization, technological innovation, and policies at different levels (i.e. local, state or national) [17]. For forest lands, the risk of conversion to other land uses is correlated with environmental, political, social, cultural, and economic factors [10, 17]. Key drivers of this conversion include changes in demographic variables [3], urban expansion [18], distance to the nearest road [10], and deforestation for commodity production [16]. Therefore, understanding the trends and long-term demographic context for population change could aid land managers and other stakeholders in mitigating the effects of residential development, especially near public lands, and anticipate future human population changes [19].

While global projections on carbon are bleak, the current situation in the US shows a better picture. Land use change at a global level is projected to contribute between 11 and 110 billion metric tons of carbon to the atmosphere by 2050 due to economic, social, and demographic trends [8]. For forest land, global trends indicate a loss in the tropics and an increase in Europe, China, and North America [14]. Specifically in the US, this trend is due to better forest management practices, reforestation, and an improvement in natural resources management, which have contributed to 11–13% of the global ecosystem carbon removal [8]. However, even though these forests are expected to continue sequestering carbon, they would do so at declining rates mainly due to aging forests and land use dynamics [20].

The US’s future in C emissions might change according to land use change projections. Some studies show that even though current estimates for the eastern US (2001 to 2012) indicate that forest land use has changed (positive or negative) less than 5%, large changes in land use are likely in the coming decades in a business-as-usual scenario [3]. For this study, recent trends indicated increasing forest areas in the southern Plains and Great Lakes’ states and losses in forest areas in the central and south-central states. Additional areas with high probability of conversion to non-forest include the Great Plains, especially in poorly stocked areas and/or sites with small diameter trees [10]. Other studies estimate as high as 36% of the land area in the conterminous United States to change in land use between 2001 and 2051 in a business-as-usual scenario [21]. According to this study, urban and forest land uses in the US are predicted to increase by 79% and 7%, respectively. On the other hand, cropland and pasture land uses are expected to decline by 16% and 13%, respectively [21].

Overall, the study of land use change is critical in forest C dynamics and better land use planning is needed to secure ecosystem services provided by forests. Even though some C stocks are increasing due to forest regrowth, especially from agricultural abandonment [22, 23], it is critical that we address the issue of forest conversion due to its significant contribution on the C budget. For example, in the US, forest lands, harvested wood products, and urban trees together offset more than 11% of the total annual GHG emissions [24]. Here, C uptake estimates for forests remaining forests were −564.5 MMT CO_2_ equivalent (eq.) in 2018. On the other hand, C emission estimates for forests which transitioned to other land uses during the same year were 127.4 MMT CO_2_-eq [24]. To help address these issues, our research focuses on modeling the relationships among disturbances, land use change, and aboveground C stocks in six US states over an 18-year period. The research objectives are to (1) model the probability of forest conversion and C stock’s dynamics using USDA Forest Service Forest Inventory and Analysis (FIA) data and (2) create wall-to-wall maps showing estimates of the risk of areas converting from forest to non-forest. We hypothesize that forests which are heterogeneous, accessible, close to urban areas, or affected by natural disturbances will have a higher rate of conversion. This research will allow us to better understand the impacts of land use change on the forest C cycle and be able to more effectively determine priority areas for management and land use planning.

## Methods

### Study area

**Fig. 1:**
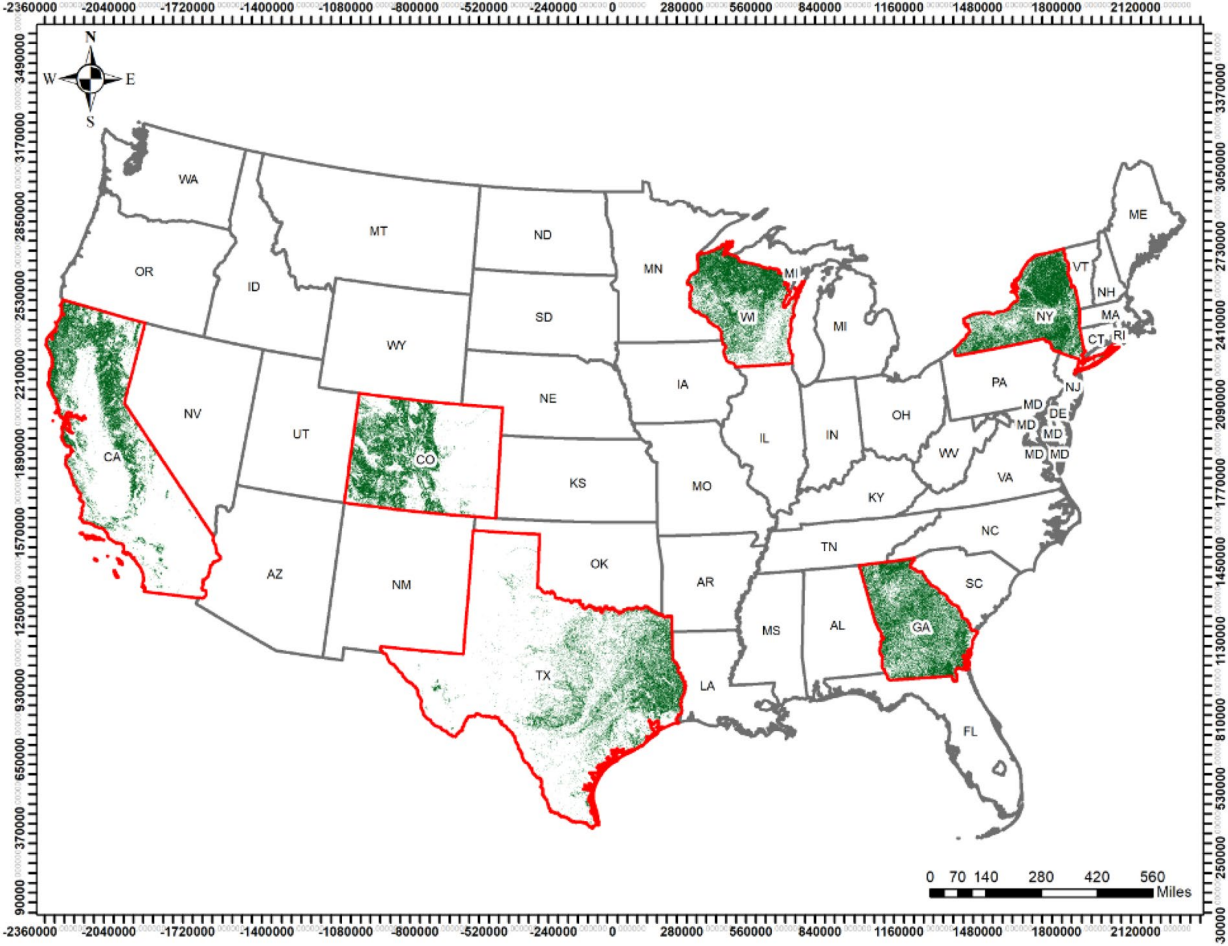
Study area across the United States displaying state boundaries. Highlighted areas show the states selected for this study. Green areas show forested areas in those states

This research includes six US states: California, Colorado, Georgia, New York, Texas and Wisconsin (Fig. 1). These six states were chosen because they are spread throughout the country, they have very contrasting ecological and socioeconomic differences, and represent each of the US FIA work units [25]. This diversity in locations allowed us to represent different forest types, disturbance history, population dynamics and drivers that could influence our study variables. In addition, there were at least two plot measurements for all of those states, which allows for a temporal analysis of change. The study area was limited to areas with forest land cover at the start of the study. For this manuscript, we consider the FIA definition of forest land, defined as land at least 0.4 ha in size and 36.6 m wide that contains at least 10% canopy cover by live tally trees of any size or has had at least 10 percent canopy cover of live tally species in the past [25].

Forests in the US cover more than one-third of its landscape and comprise more than 28 billion cubic meters of wood volume. Even though overall forest area has remained stable, there have been changes at regional and local scales that are not reflected by aggregated numbers. In addition, the road network in the country has expanded, making forest lands more accessible to people [26]. This easier access to forests would likely encourage land use changes. Major natural forest disturbances nationally include wildfire, insects, and disease [26]. These and other disturbances, as well as changing forest conditions, cause changes in tree species composition and distribution. Climate in the study region varies widely.

### Data collection

Ground data used in this study were collected and organized into a database by the USDA Forest Inventory and Analysis (FIA) program from 2000 to 2017. This database includes different phases and tables depending on the scale and level of detail of the information contained in each. For this research, we worked with ground-sampled FIA plots that were remeasured approximately every five years for eastern states and 10 years for western states, which allowed for a spatiotemporal analysis. These plots covered a 0.4 hectare sample area and were divided into four fixed-radius (7.32 m) subplots. Within each subplot, a microplot (2.07 m radius) was nested. Here, trees with a diameter at breast height (d.b.h.) less than 12.7 cm but greater than 2.54 cm were measured. Forest and non-forest conditions were assigned by the field crew in each remeasurement, which allowed us to track any land use changes that occurred. To obtain human population and housing information, we used the United States’ 2010 National Census [19]. For obtaining spectral information, we used a mosaic of Landsat 8 surface reflectance imagery (30 m spatial resolution) from the period 2018-05-01 to 2018-09-30 (which corresponded to the summer months) obtained from the Google Earth Engine platform (LANDSAT/LC08/C01/T1_SR). The initial image collection consisted of 4093 Landsat images that covered the study region during the summer months.

### Data analysis

Information at the subplot and microplot levels was gathered for the different time periods available (initial time *t*_*i*_ and final time *t*_*f*_) and scaled-up using the FIA expansion factors to a plot level (minimum modeling unit). The initial data set was divided into training and test data sets to fit and validate the models. The total number of plots with complete observations was 11,262, which where subdivided into the training and test data through probability samples with replacement in R (using a probability value of 0.7 for the training data and 0.3 for the test data) [27].

Our goal was to build one model per variable of interest (response variable). Therefore, model comparisons were performed to select the model which yielded the optimal fit statistics. Because our end goal was to build a replicable model applicable across the continental US, we included the variables state and forest type in both the LUC and the C change models. This allowed us to account for and capture the differences between the six states that were not captured with the rest of the explanatory variables.

The response variable for the logistic model was the probability of forested areas being converted to mixed or non-forested areas. For the purposes of this study, we will refer to forested plots which transitioned to mixed (partial forest/non-forest plot, e.g., one forest plus one non-forest condition) or non-forest conditions as plots that changed in land use. The response variable for the C model was C stock change in the total aboveground live biomass (defined as the sum of dry biomass located in the merchantable bole, top and limbs, stump, woodland tree species and saplings).

#### Model building

To explore the performance of different modeling approaches, we compared two main methods: a statistical model (parametric approach) and a machine learning algorithm (non-parametric approach). We compared the performance of these two main approaches to select the best performing model for each variable of interest. For both models, parametric methods were superior in predicting our response variable (See Additional file 1 for the Random Forests results and background). For the statistical models, we used mixed effects modeling because we worked with ecological data that had nested data, as well as temporal and spatial correlation structures. To select which type of mixed effects models to use, we looked at the characteristics of our variables of interest, especially the response variable. For the land use change model, the response variable had a binary behavior (0: no change and 1: change). Therefore, we chose a logistic model, which would give us the probability (or odds) of the response variable. In addition, for the C change model, the response variable was quantitative and had a linear relationship with the predictor variables. Therefore, we selected a linear regression model that accounted for correlated data, a linear mixed effects model with a random intercept.

##### Generalized linear mixed effects models

The generalized linear mixed effects models encompass several different types of regression models (i.e. general linear models, logistic regression, Poisson regression) while accounting for spatial and temporal correlations and nested data. This family of models has two main components: fixed effects (which includes the variables we are interested in) and random effects (which acknowledge the effect of the variables included here with a variation that is normally distributed with a certain variance) [28, 29].

For both the LUC and C change models, the training data were used to create a generalized mixed-effects model for each of the response variables. Explanatory variables (covariates) for both models were divided into the following categories: (1) forest attributes (ecological variables such as trees per hectare, basal area, and ecoregion), (2) plot attributes, (3) topography, (4) census data, (5) forest disturbances, and (6) number of conditions present in a plot (e.g., number of forest and non-forest types differentiated by reserved status, owner group, forest type, and stand density). The number of conditions present in a plot gave us a good representation of heterogeneity in a plot and allowed us to work with single- and multiple-condition plots. We regrouped the ecological classification codes into broader ecoregions due to the large number of categories, which would have complicated the model. See Additional files 2 and 3 for an extensive list of the variables used and metadata.

Variable selection techniques were applied in two stages. The first stage included dividing the variables into the categories defined above. Within each category, Pearson correlation coefficients were calculated to quantify relationships between quantitative variables. Variables with a coefficient of 0.5 or higher were marked to later verify that no correlated pair were kept in the final model. Categorical variables were visually assessed through scatterplot matrices. No variables were discarded in this step. In addition, for the logistic model we used the information value (IV) indicator to see how strong of a predictor each variable was and in which order to include the variables in the model (Additional file 2). The IV is used to select important variables in a logistic predictive model by ranking them based on their importance. We calculated the IV with the create_infotables and IV functions in R [30].

Variables were added to the model one at a time in descending order of their IV level of importance. For the regression model, variables were incorporated into the model according to the variable importance ranking obtained from the random forest model (Additional file 1). For both models, the Akaike information criterion (AIC) was used for model comparison. If an added variable increased the AIC value, it was removed. After this first variable selection stage, variables kept in each category were merged back to proceed with the variable selection.

**Fig. 2:**
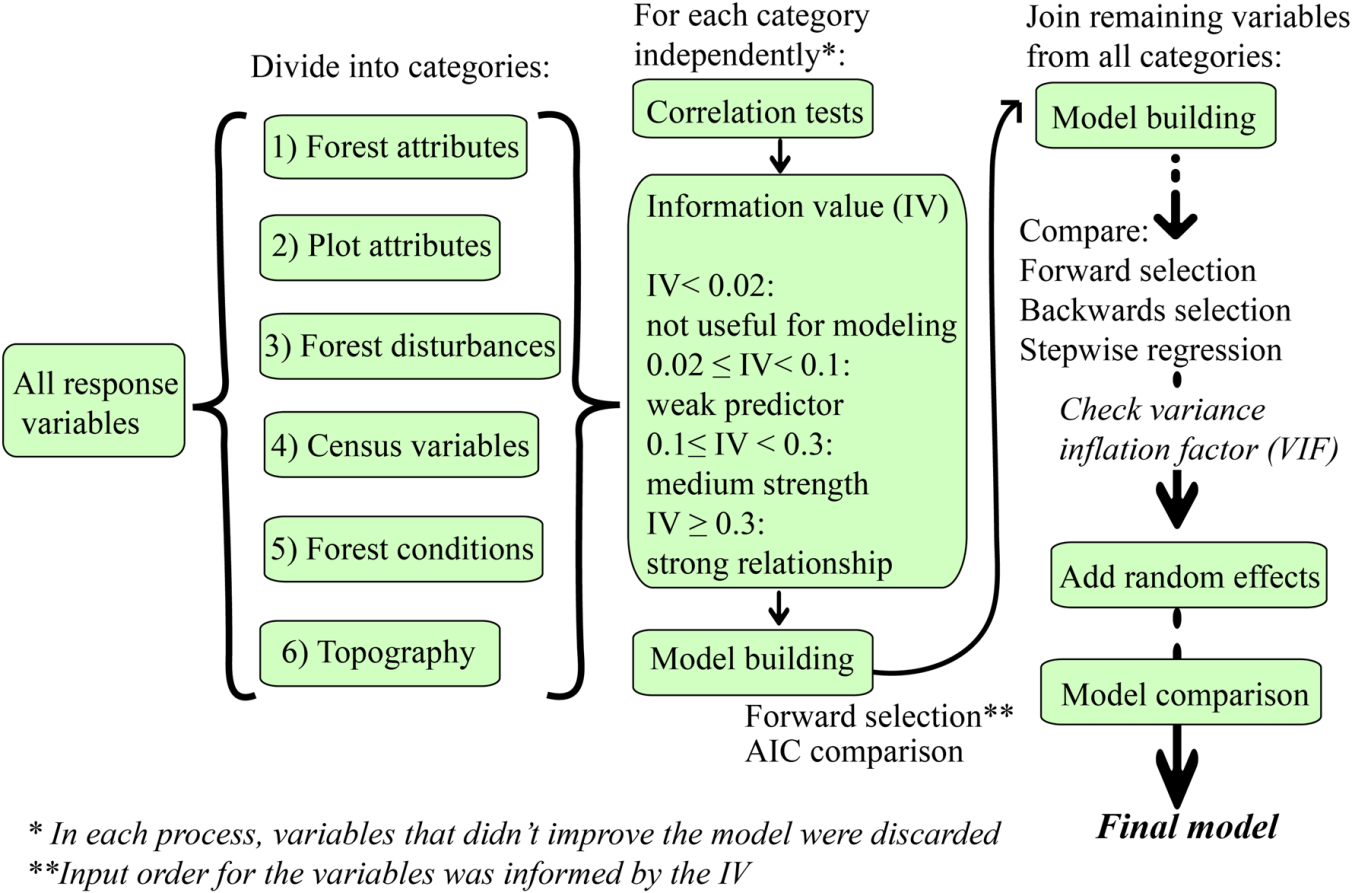
Workflow for the variable selection process to determine the probability of forests becoming non-forested (logistic model) and aboveground C stock changes (linear mixed-effects model)

We compared outputs of three variable selection approaches in R software using the car [31] and MASS [32] packages: forward selection, backward selection and stepwise selection. Finally, we used the variance inflation factor (VIF) to verify there was no multicollinearity (VIF > 10) present in the model. A logistic regression model with the remaining variables was created for the land use change model using the glmmPQL function in R with the *cloglog* link [32]. In contrast, a linear mixed-effects model was implemented for the C model using the glmer function [33]. For both models, different combinations of the retained variables plus state and forest types or ecoregion (independently and nested within each other) in the fixed or random section of the model were validated with the test dataset. This was done to account for correlated data points in our spatiotemporal analysis. In addition, interactions between potentially related covariates were examined. A complete workflow of the process can be observed in Fig. 2.

#### Model comparison

For both generalized mixed-effects models (logistic and linear models), a comparison between these techniques and the random forest approach was done to identify the best performing model. The criteria used to select the model was to look at the percentage of omission and commission errors, the overall accuracy, precision, and recall values. While we used the AIC values to compare models at the initial stage (to filter out preliminary models, Fig. 2), once we incorporated random effects in the models, AIC values were no longer estimated. Since we used the glmmPQL function in R to compute the models, no quasi-likelihood can be estimated for these mixed-effects models [34]. In the case of the C model, the root mean square error (RMSE) was used for model selection.

#### Creating predicted wall-to-wall likelihood of forest change

**Fig. 3:**
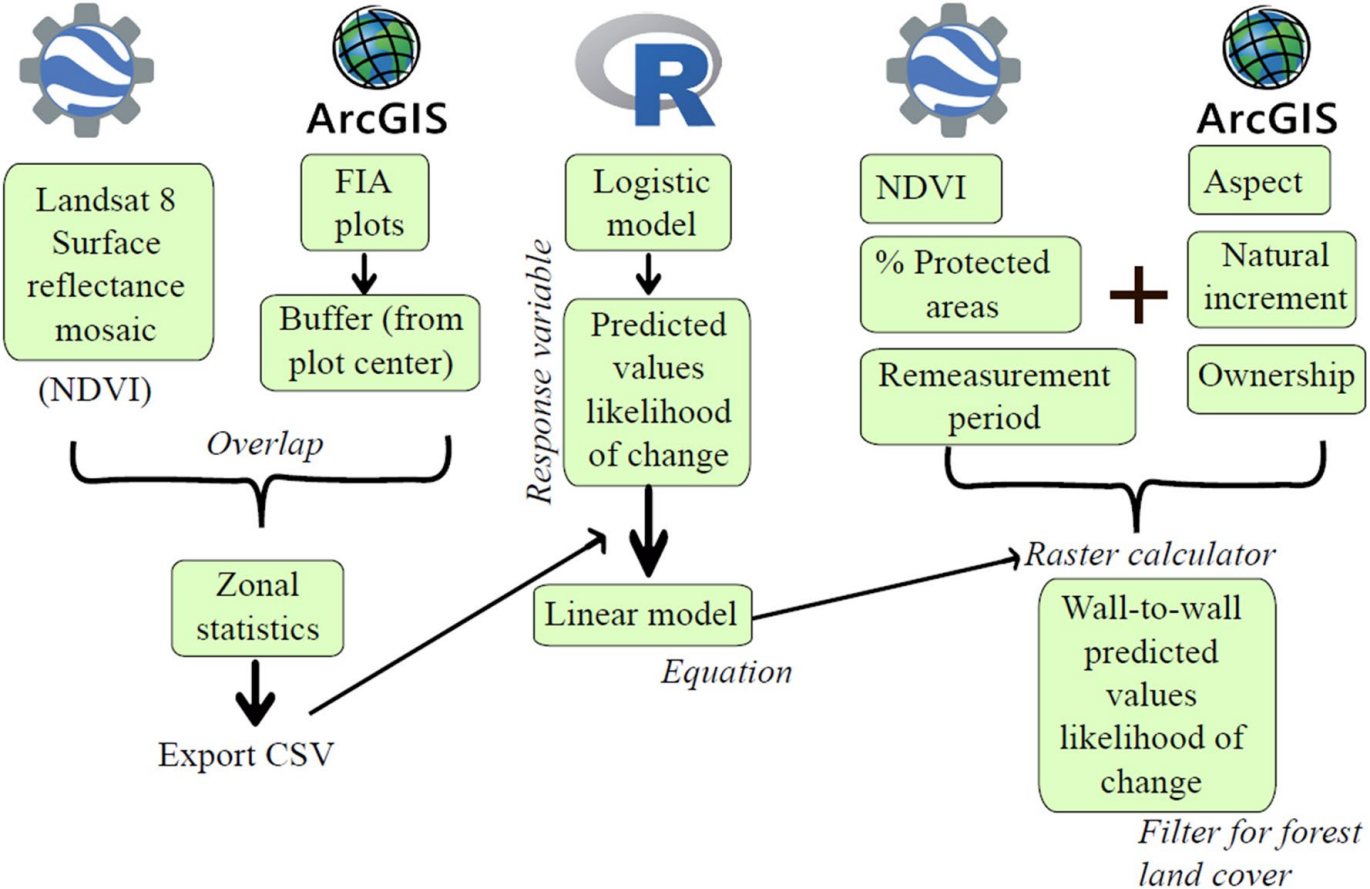
Step-by-step workflow for calculating the wall-to-wall likelihood of forest land use change for the study area showing processes in both ArcGIS and R environments and data layers used in each

We created a spatially continuous map that covered the entire area of the six states of interest in a pixel by pixel format (i.e., a wall-to-wall map). This wall-to-wall map used the FIA data and the logistic model equation (See "Model building" section) to predict the likelihood of conversion of forested areas for every pixel. We used only the explanatory variables from the logistic model that had information available at a wall-to-wall scale to build the maps (See Fig. 3 for variables kept in the map and Additional File 2 for the complete list of variables). These variables were obtained through Google Earth Engine (GEE) and ArcMap 10.8 using original FIA coordinates, mosaic Landsat 8 images, and digital elevation data [35]. The variables used were: Normalized difference vegetation index -NDVI- (obtained from the Landsat 8 mosaic) as an estimate of basal area, ownership code [36], aspect [35], remeasurement period (Mean value per state from the FIA plot data), percentage of protected area in a county [19], and natural increase of the population (between 2000 and 2010) [19]. State and county boundaries were obtained from the United States Census Bureau [37]. All layers were converted to a raster format and transformed to the same projection (WGS84).

The NDVI variable was calculated in the GEE platform. Bands 5 and 4 from the imported Landsat 8 Surface Reflectance images for the summer months of 2018 for each state were used to generate a maximum NDVI for each pixel. This was done to obtain a mosaic of pixels displaying the maximum NDVI value for the period selected which would avoid clouds and ensure a value where hardwood trees had leaves. To extract NDVI and aspect values for each pixel, we used true FIA plot coordinates. If more than one plot measurement was available over time, means for plot coordinates values were calculated to account for variability and errors in each coordinate measurement. A buffer of 180 feet was used around the plot center to replicate the extent of a plot and calculate mean values for each pixel through zonal statistics.

With this wall-to-wall information, a multiple linear regression model was fit in R (with the predicted probability of change previously calculated as a response variable, see “Model building” section), obtaining an equation for the linear model. This equation (see Additional file 2) was used with the raster calculator tool in the ArcGIS environment. The maps generated here were clipped with the National Land Cover Database (NLCD) to display only forested areas. This new raster layer displayed the probability of conversion from forest to another land use (See Fig. 3 for a visual workflow).

For interpretation of the results shown in the maps, we overlapped ownership [36], protected areas [38], digital elevation models (DEM) [35], and major cities' shapefiles [37] to our maps.

## Results

**Fig. 4:**
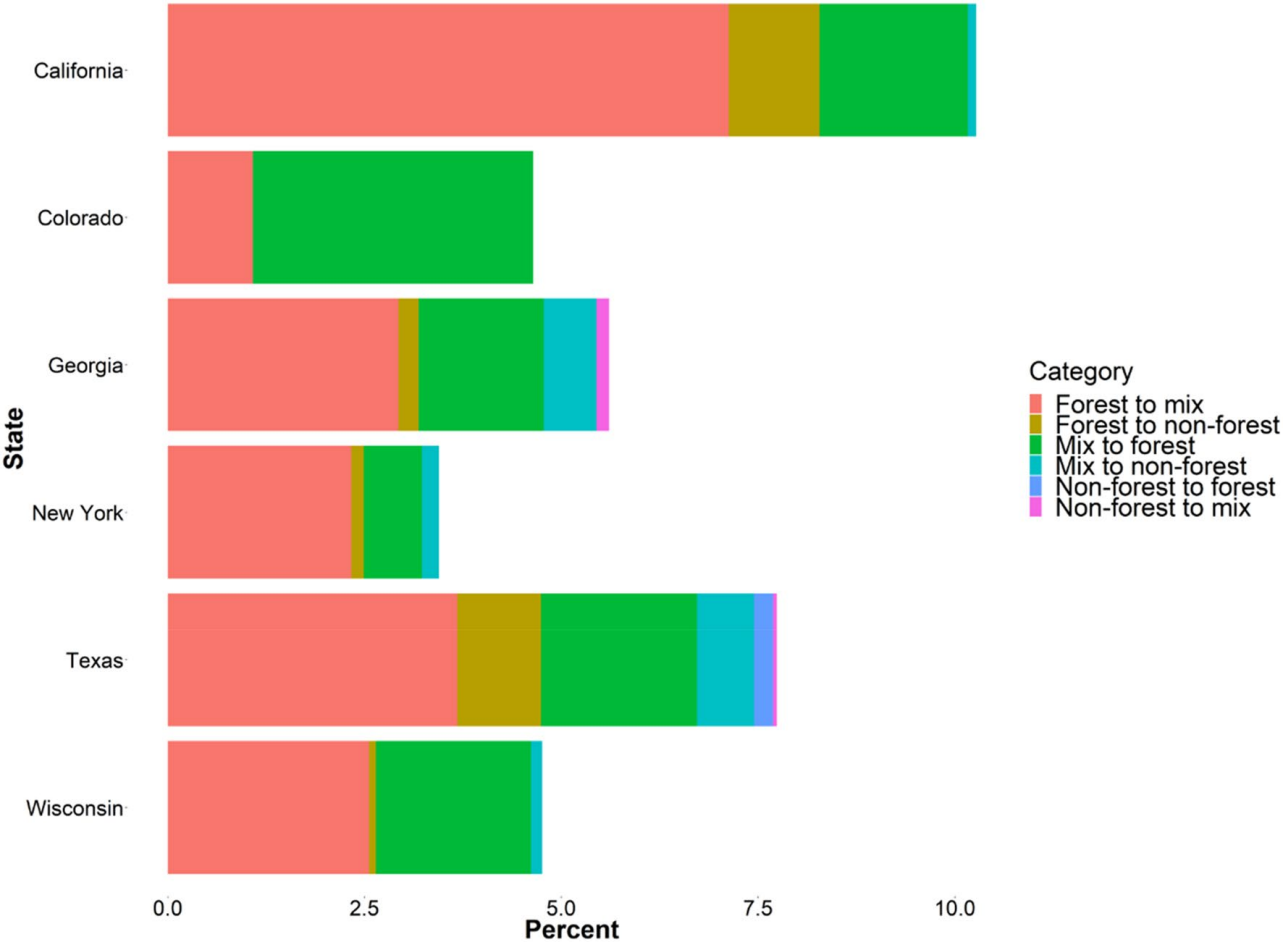
Percent of FIA plots across six US states that have transitioned to and from forested conditions in the study area between 2000 and 2017

The initial exploration of the FIA data for the six states revealed some important foundational knowledge. From 2000 until 2017, California, Colorado, New York, and Wisconsin had an increase in mean aboveground live biomass, while Georgia and Texas had a mean reduction in live biomass. On the other hand, mean biomass for standing dead trees increased in California, Georgia, New York, and Wisconsin (Table 1). We also observed that 3.4% of forested plots transitioned to mixed or non-forested conditions and 0.7% of the plots underwent a complete land use change (to non-forest). California had the highest percentage of plots that changed in land use, followed by Texas (Table 1 and Fig. 4).

**Table 1:** Estimates for aboveground biomass and change from forest land use, FIA data

State	Change in aboveground live biomass (Mg/ha)	Change in aboveground dead biomass (Mg/ha)	Number of plots evaluated	Percentage of forested area (%)	Number of plots that transitioned from forest to mix or non-forest condition	Percentage of plots that transitioned per state
California	0.36 ± 2.71	0.72 ± 5.14	954	21.38	79	8.28
Colorado	0.72 ± 2.44	− 0.98 ± 3.20	646	28.49	7	1.08
Georgia	− 0.02 ± 1.18	0.54 ± 7.88	3139	58.81	100	3.19
New York	0.05 ± 1.13	0.78 ± 4.19	1887	56.34	47	2.49
Texas	− 0.05 ± 1.16	− 0.26 ± 7.65	2067	12.99	98	4.74
Wisconsin	0.06 ± 0.98	0.49 ± 3.93	5632	42.18	149	2.65
All states	0.38 ± 5.73	0.08 ± 1.35	14,325	26.94	480	3.35

Mean estimates (± standard deviation) and plot information for the variables of interest across six states in the US, 2000–2017

### Logistic regression model—forest change

Out of the 32 initial variables (Additional file 2), nine were retained after the selection process for fixed effects: basal area live, basal area dead, remeasurement period, ownership code, percentage of protected area per county, natural increment per county, number of forested conditions in a plot, number of non-forested conditions in a plot, and aspect. For random-effects, forest type was a stronger predictor than ecoregion (IV of 0.52 compared to 0.35 for ecoregion) (Additional file 2).

**Fig. 5:**
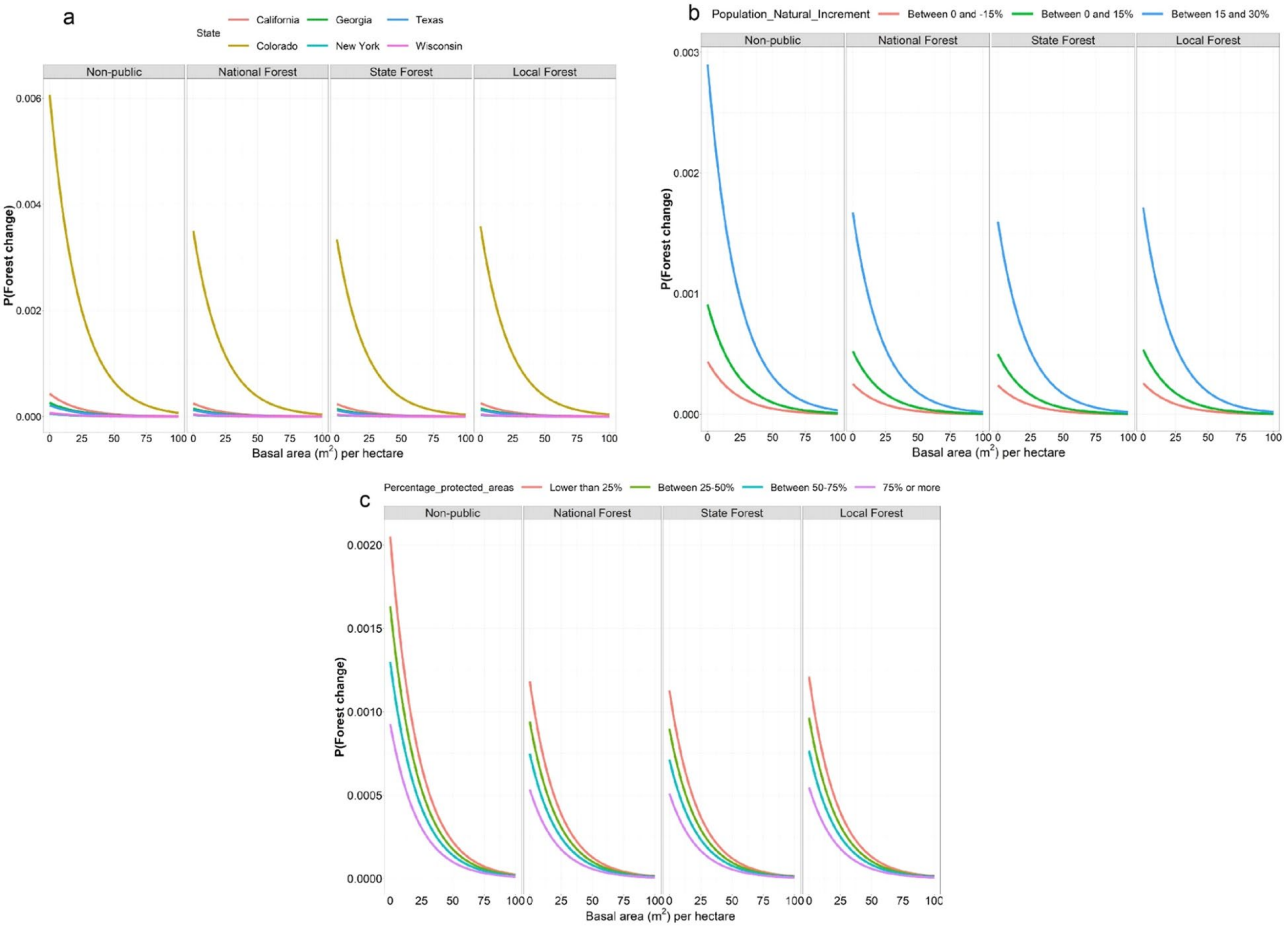
Predictions from the logistic regression model. Response variable is the probability of forest land use change to mixed or non-forest conditions. The four horizontal panels show different land ownership classes. **a** The probability of land use conversion for the six states analyzed. **b** The effects of population natural increment. **c** The effects of the percentage of protected area in a county

Results from the validation of the logistic model show that class 0 (no forest land use change) had a very low omission and commission error (between [0.05–0.02] and [3.26–3.33], respectively) (Table 2). However, for class 1 (change from forest to other land use), the omission error was high (around 98%). This might have been due to the model having difficulty capturing class 1 change because there was a higher proportion of FIA plots that did not change in forest land use between *t*_*i*_ and *t*_*f*_, compared to the number of plots that did change. Overall accuracy was over 96%, which should be interpreted with caution as it might be driven by the greater proportion of correctly classified 0 s. Comparing all these indicator variables, the logistic regression was selected with forest type nested within state as random effects. Coefficients and equations for the final model can be observed in Additional file 2 and predictions in Fig. 5.

**Table 2:** Model comparison between random forest and the final candidates for the logistic regression models (Land use change model)

Model	Accuracy	% Omission	% Commission	Class	Precision	Recall
Random forest	96.589	0.05	3.37	0	0	0
		100	100	1	100	1
Logistic model						
State as a fixed effect	96.68	0.02	3.3	0	71.43	1.48
		98.52	28.57	1	28.57	1
Forest type as a fixed effect	96.7	0.03	3.27	0	72.73	2.37
		97.63	27.27	1	27.27	1
State as a random effect	96.68	0.02	3.3	0	71.43	1.48
		98.52	28.57	1	28.57	1
Forest type as a random effect	96.69	0.03	3.28	0	70	2.08
		97.92	30	1	30	1
Forest type nested within state as a random effect	**96.71**	**0.03**	**3.26**	**0**	**75**	**2.67**
		**97.33**	**25**	**1**	**25**	**1**

Bold text indicates the selected model. Category 0 indicates no change from forest land use, while class 1 indicates a change from forest land use

Colorado had the highest probability of forest conversion compared to the other five states followed by California. Areas with high population natural increment and the lowest percentage of protected area had a higher predicted probability of conversion. In addition, non-public areas had a higher probability of conversion to non-forested areas (Fig. 5).

### Mixed-effects linear model—aboveground live carbon change

Out of the 36 initial variables (Additional file 3), after the selection process for fixed effects, nine variables were kept: basal area live, basal area dead, trees per hectare live, distance to roads, disturbances (simple/compound), percentage of protected area per county, number of forested conditions in a plot, condition at *t*_*f*_, and change from forest to other land use. Different combinations of linear models with these variables plus state and forest types or ecoregion in the fixed or random section of the model were validated with the test dataset. Forest type was a stronger predictor than ecoregion when comparing AIC; hence, forest type nested within state was used as the random effect.

Coefficients and the equation for the final model can be observed in Additional file 3. The mixed-effects model had a lower root mean square error (RMSE) compared to the random forest model (12.06 compared to 13.56 Mg/ha); hence, the mixed-effects model was the one selected to predict change in aboveground live C stocks. The bias for this model was − 0.899 Mg/ha.

**Fig. 6:**
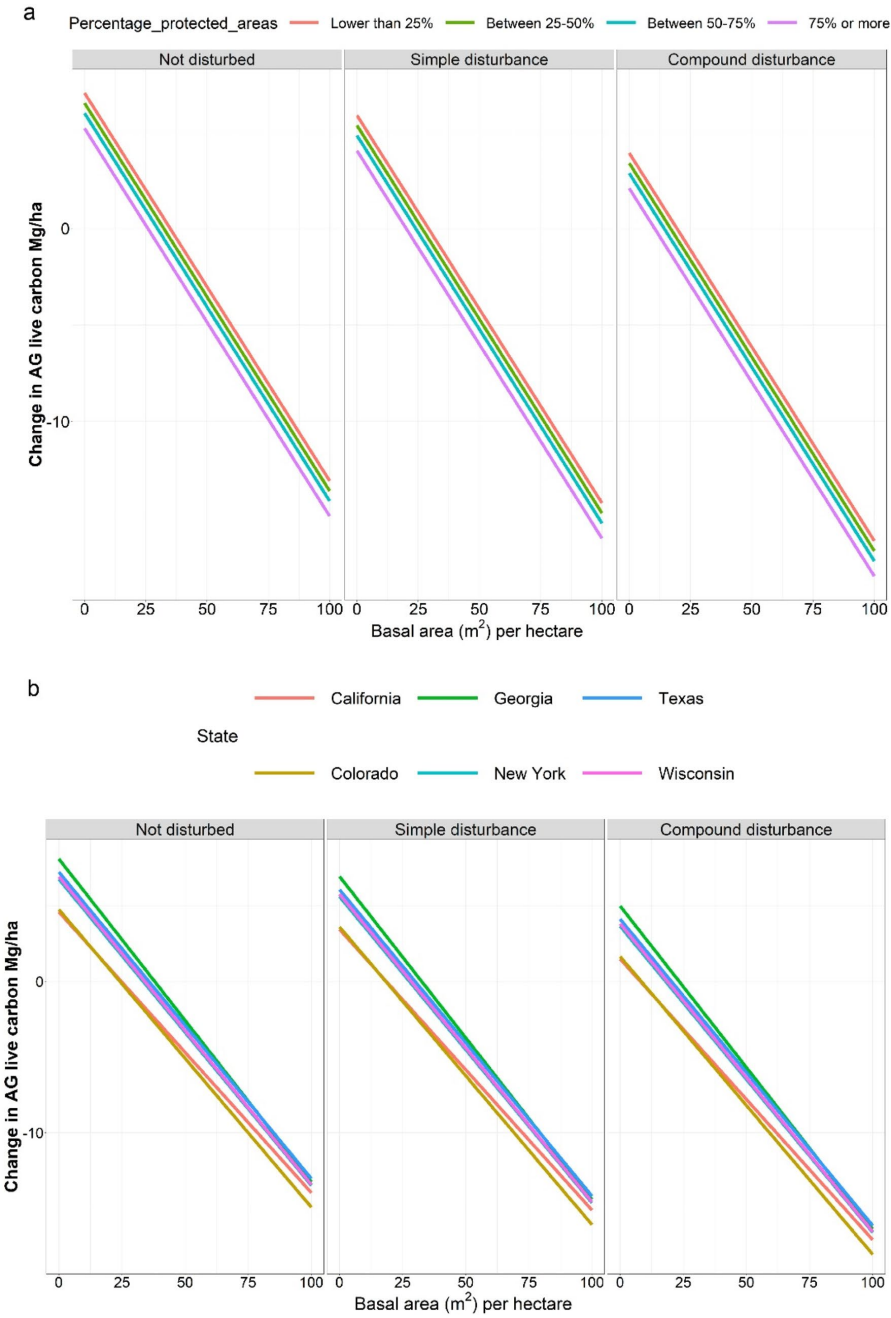
Predictions showing the change in aboveground (AG) live C stocks. The three horizontal panels show different disturbances classes. **a** Shows the change in C for the six states analyzed. **b** Shows the effects of the percentage of protected area in a county

Colorado and California had a more negative change in aboveground live C compared to the other states (Fig. 6). Areas with the lowest percentage of protected area had a more negative change in C. In addition, disturbances had an effect on the aboveground C stocks. We observed that plots with more than one disturbance in between remeasurements (compound disturbance) had a more negative change in C than plots being affected by one (simple) disturbance.

### Creating predicted wall-to-wall likelihood of change

**Fig. 7:**
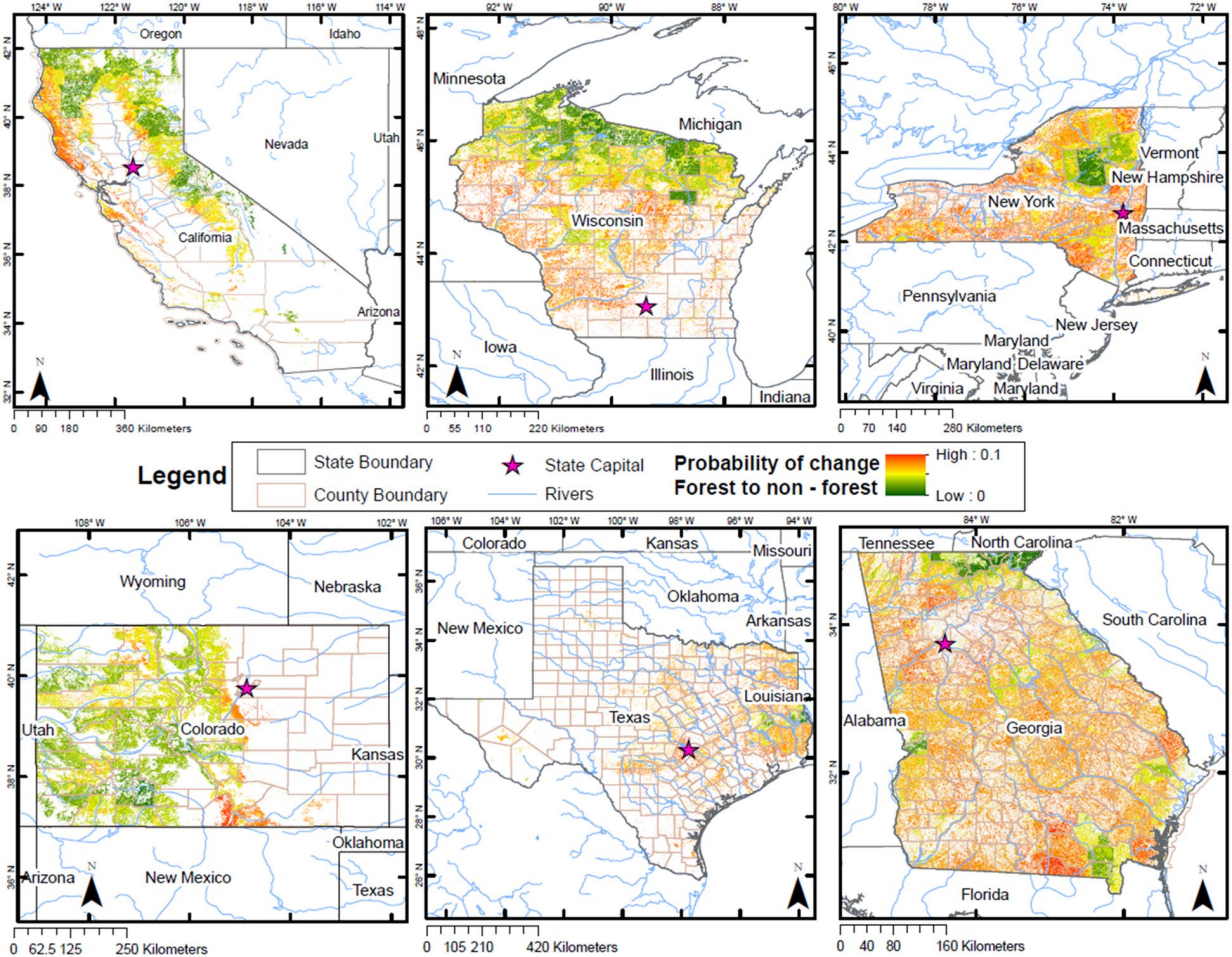
Wall-to-wall maps of the probability of forest areas to convert to non-forest. Greener areas show less probability of conversion, while yellow areas represent a moderately low probability (< 0.05), orange areas represent a moderately high probability (0.05–0.075), and dark orange-red colors show a higher probability of conversion (0.075–0.1). Probability values range from 0 to 0.1

Public areas (e.g., forests managed by federal and state agencies) and protected areas within the six states have a lower probability of conversion to non-forest (Fig. 7). Second, we see that areas closer to big cities and coastal areas tend to have a mid to high probability of conversion. Third, topography plays an important role, in that higher elevation areas where accessibility is difficult are represented by areas that have a low probability of conversion to non-forested areas (e.g., mountain ranges). Fourth, in most of the states, areas closer to rivers tend to have greater risk to convert to non-forested areas (moderately high to high probability).

From the wall-to-wall maps, we observe that California had a mean probability value for forest conversion of 0.05 ± 0.02. California's natural protected areas such as federally-managed forest lands (e.g., Six Rivers, Shasta Trinity, Sierra National Forests, and Yosemite National Park) displayed a low probability of conversion (mean: 0.0445 ± standard deviation: 0.0135).^1^ The Sequoia National Forest (0.0575 ± 0.0055) had a moderately high probability of conversion to non-forest. This area has had a very high natural increment in its population. In addition, coastal areas exhibited a moderately high probability of conversion, while mountain areas a low probability.

The mean probability value for forest conversion for Colorado was 0.039 ± 0.012. Compared to other states, areas surrounding rivers in Colorado showed a low probability of conversion. This area is characterized by an abrupt topography, similar to mountain ranges, which also have a lower probability of conversion. Colorado’s natural protected areas such as Rio Grande National Forest, which has very few small towns in its surroundings, presented a low probability of conversion (0.0299 ± 0.0095). However, the Colorado Springs area had a moderately high probability of conversion to non-forest (0.0598 ± 0.0083). This area had a high natural increment in its population. Costilla and Las Animas counties displayed a high probability of conversion (0.0726 ± 0.0067 and 0.0635 ± 0.0008 respectively). There was a low percentage of protected area in these counties as well as more private than public forest lands. Concerning modeled C stocks, both California and Colorado showed separation from the rest of the states (following the same trend as the logistic model of forest conversion) and showed a more negative net change in aboveground C stocks.

Georgia had a mean probability value for forest conversion of 0.042 ± 0.012. In Georgia, areas around the Appalachian Mountains represented a low probability of conversion, as well as protected areas such as the Okefenokee National Wildlife Refuge (0.0185 ± 0.0071). Areas surrounding the city of Atlanta had a moderate to high probability of conversion, as well as areas surrounding rivers. The state is characterized by a low percentage of protected area per county and a high natural increment of its population in most counties.

New York had a mean probability value for forest conversion of 0.029 ± 0.015. Forest ownership in New York is similarly distributed between private and public areas, making this an important variable that drives forest conversion in the state. Protected areas such as the West Canada Lake Wilderness and the High Peaks Wilderness presented a low probability of conversion (0.00 ± 0.0083 and 0.0106 ± 0.0093 respectively). In addition, areas like the Big Indian Wilderness and Allegany State Park had a low probability of conversion as well (0.0250 ± 0.0093 and 0.0218 ± 0.0119), similar to areas federally owned. In general, there was a low percentage of protected area in the state.

Texas had a mean probability value for forest conversion of 0.042 ± 0.011. Coastal areas in Texas and areas close to major cities like Austin, Dallas, Houston and San Antonio had a higher risk of conversion. The Big Thicket National Preserve Area (0.0281 ± 0.0019) and the Sabine National Forest (0.00 ± 0.0050) area presented a low probability of conversion. The eastern border of the state (where pine forests are located) was predicted to remain as forests. Areas that surrounded big lakes and cities showed a moderately high probability of conversion to non-forest.

In Wisconsin, the mean probability value for forest conversion was 0.019 ± 0.016. An important result that stands out in Fig. 7 is the dark green and well-defined area that makes up the Menominee Reservation. This area was predicted to remain as forest, as represented by its low probability of conversion (0.00 ± 0.0054). Moreover, protected areas such as the Black River State Forest (0.0090 ± 0.0060), the Apostle Islands (0.00 ± 0.0055), and the Chequamegon National Forest (0.0018 ± 0.0080) displayed a low probability of conversion. Similar to other states, areas close to the Great Lakes (e.g., Lake Michigan), rivers (e.g., the Mississippi and the Wisconsin Rivers), and major cities (e.g., Milwaukee) have a higher risk of conversion. Summary data for each state including the area predicted to convert per probability bin is displayed in Table 3.

**Table 3:** Summary table from the wall-to-wall map predictions showing the area and percentage that each probability bin represents per state

Probability Bin	0–0.025		0.025–0.05		0.05–0.075		0.075–0.1		Total area (sq km)
State/Variable	Area (sq km)	(%)	Area (sq km)	(%)	Area (sq km)	(%)	Area (sq km)	(%)	
California	20.797	2.66	355.441	45.44	330.452	42.24	75.579	9.66	782.269
Colorado	64.450	8.25	586.057	74.99	125.249	16.03	5.713	0.73	781.468
Georgia	87.175	10.54	526.238	63.65	213.359	25.81	0.000	0.00	826.772
New York	233.904	34.75	429.178	63.75	10.101	1.50	0.000	0.00	673.182
Texas	64.023	7.19	649.975	72.98	176.652	19.83	0.012	0.00	890.662
Wisconsin	429.514	60.86	275.060	38.98	1.155	0.16	0.000	0.00	705.729

## Discussion

Our results show that land use change affects forested areas, especially due to growing populations and urban development. Urbanization is a main driver of the loss of forests in the US [16, 18], which also brings agricultural expansion. Other studies specifically report demographic variables [3] and proximity of roads [10] or rivers as important urbanization variables that affect forests. River valleys tend to have a flat topography, provide better accessibility, and have a higher population density that surrounds them. Given the increase in housing relative to population growth in the US [19], demographic variables are critical in estimating forest conversion and C emissions. Through the application of the C model, we observed that forest conversion affects C fluxes, especially at large scales. This relationship between land use change and C fluxes has been reported in other studies [2, 3, 5–7]. Therefore, it is critical to incorporate estimates of land use change in C studies and C in land use change studies.

Although we observed that less than 4% of the FIA plots transitioned from forest to other land uses, prior studies [3] have shown that large changes are likely to occur in the coming decades in a business-as-usual scenario in eastern US forests. Similarly, around 36% of the forested area in the conterminous US area is estimated to change in land use [21]. Other studies [10] provide regional mean values for forest conversion which are higher than the ones we obtained. For example, for the Lake States region, this study shows a 0.086 estimated probability compared to our value of 0.019 for Wisconsin; for the Southern region, 0.082 compared to the 0.042 that we obtained for Texas, for the Southeastern region 0.084 compared to our value of 0.042 for Georgia, and finally, the Mid-Atlantic region had a value of 0.076 compared to our value of 0.029 for New York. Therefore, it is key to identify critical regions that have a higher probability of conversion, despite the actual probability being low relative to other risks.

An important result of this research is that demographic variables (such as the population natural increment), ownership of the land, level of protection, and live and dead biomass (represented through basal area and trees per hectare) represent important factors driving forest conversion. With respect to ownership of the land, motivation to sell land or change its use might play an important role in private lands [39, 40], as well as short-term economic factors and technological innovation [17]. These reasons could explain the differences in probability of conversion observed in this study among ownership groups.

Disturbances were not a major factor when modeling the probability of forest conversion for all the states together. Independently however, California and Colorado had a stronger influence of the forest disturbances variable. This may be due to widespread insects, diseases, and fire [41]. Even though the disturbance variables were not kept in the model, their influence in these states might be captured by the state coefficients (used as random effects). These disturbances might explain the difference observed in Fig. 5a, in which both states show a higher probability of conversion compared to other states. On the other hand, when estimating aboveground C stock changes, the presence of disturbances decreased C fluxes whether those disturbances were simple or compound.

Our logistic regression approach provided a straightforward prediction of land use change that assigned a land use change probability across our study region. Land use change is a stochastic process that is difficult to model because transitions are rare and independent from one another and depend largely on the time period observed between remeasurements. Zero inflated models may be useful in future applications; however a zero-inflated model is used to account for an elevated number of zeros (more than we were to expect) [28], but this was not the case with our data. The FIA program evaluates plots on forested lands which are most likely going to remain forests where only a few would be expected to transition to another use. In addition, the binomial family (which encompasses logistic regression) allows binary responses in the response variable [28]. The level of detail and scale of each covariate is also important as it will determine the detail of reporting. For example, due to the level of detail of the census variables included in the model, some of the county boundaries shown in Fig. 7 influence the pattern shown in the map. This is not necessarily the case for the Menominee Reservation, which has conducted intense forest management [42–44] which is reflected in Fig. 7. When predicting carbon stock changes, parametric models do well in providing quantitative predictions and can be modified to reflect changes in forest conditions, such as changes in forest types, species composition, and climate.

It should be noted that our analysis began with using inventory data from forested plots. While understanding land use change in forestry was the primary motivation of this study, including additional data sources, such as other remote sensing data products [45, 46], could help us to better understand transitions across all land uses. Incorporating additional remote sensing products can help to build spatially continuous maps that can aid managers and policymakers in identifying regions at risk of transition land use. Future analyses could integrate remote sensing with other modeling approaches to determine the spatial extent of land use changes. Relying on quality input data as obtained through national forest inventory data can help to better understand changes across forested landscapes.

## Conclusions

Land use change and resulting contributions to the global C budget are accelerating. A better understanding of the drivers of land use change is needed to reduce the loss of forested areas and C emissions, as well as to increase the resistance and resilience of forests. From our study, we saw that the main drivers of forest conversion in FIA plots were the live and dead biomass present, the amount of protected land, the natural increment of the population, and ownership of the land. An important finding was that disturbances were not a strong predictor of forest conversion, except in California and Colorado where disturbance effects might be captured through the state coefficients. Moreover, resiliency of aboveground C stocks mainly depended on the live and dead biomass present in the forest and its structure, the elevation of the area, the disturbance history, and the area under protection.

Our results will help policy-makers prioritize forest management activities and land use planning by providing tools and spatial information that will help them create forest management strategies that enhance forest health and productivity. Those strategies could include promoting extension programs that reinforce the value of forest, provide economic incentives towards forest management, and implement new technology for monitoring and harvesting resources. These programs should be specially targeted to landowners.

Moreover, to mitigate climate effects on forests, this research highlights priority areas where more intervention is needed to keep these areas as forest despite the changing conditions and increase in disturbances. This research will directly assist six US states (CA, CO, GA, NY, TX, and WI) and if expanded, the benefits can be applied to the entire United States or even worldwide because of the scale at which land use change problems occur.

Overall, this study confirms that land use change is associated with C fluxes and that the zoning of urban and agricultural areas is key to avoid an increase of C emissions that contribute to climate change. Population growth creates an inevitable need to dedicate more areas for residential and agricultural purposes. However, through more focused forest management that increases the resistance and resilience of ecosystems and prioritizes critical areas, forest health, and productivity, we can create strategies to balance population and ecosystem needs.

## Supplementary Information

**Additional file 1.** Random Forest Model.

**Additional file 2.** Logistic regression model for land use change.

**Additional file 3.** Linear mixed-effects model for aboveground carbon changes.

**Additional file 4.** Wall-to-wall maps of the probability of forest areas to convert to non-forest separated by state.

## Data Availability

The data that support the findings of this study are openly available in the USDA Forest Service FIA Datamart public data repository at: https://apps.fs.usda.gov/fia/datamart/

## Footnotes

1 For the rest of this section, numbers in brackets will represent mean ± standard deviation of the probability value.
