# Supplementary material for: Modeling land use change and forest carbon stock changes in temperate forests in the United States
Source: Carbon Balance Manag. 2021 Jul 3;16:20. doi: 10.1186/s13021-021-00183-6 (PMC8254905; doi:10.1186/s13021-021-00183-6)
Supplement: Supplementary file 1 — Additional file 1. Random Forest Model. [file 13021_2021_183_MOESM1_ESM.docx]

**Additional file 1:**

**Random Forest Model**

For both models (land use change and C), our first approach was to use the random forest machine learning algorithm. This algorithm consists of building multiple decision trees (collectively called a forest), each of which will work with a random sample of the data. To avoid bias due to outliers, the random forest algorithm will take the average prediction from all the decision trees (i.e., the mean for regression and the mode for classification) by adopting a bagging approach^[[1]](#footnote-1)^. We opted to use a random forest algorithm due to its capability of working with a large dataset and variables of interest, as well as its capability of working well for both categorical (through classification procedures) and quantitative (through regression procedures) variables^[[2]](#footnote-2)^. The random forest algorithm was used for both response variables: change in C stocks and land use change.

We used the randomForest function in R^[[3]](#footnote-3)^ to run 500 trees and create a model with all the explanatory variables initially selected (including ecological, topographical and census variables). The advantage of this algorithm is that it can work with an extensive amount of categorical and quantitative predictor variables. For the land use change model, as the response variable was categorical, the algorithm predicted a classification. For the C model, a quantitative response was estimated. We then identified the importance of each variable in the model. For the land use change model, a confusion matrix was created to determine the ability of the model to capture the conversion to and from the different categories.

**Random forest for land use change**

For complete names of the variables, please see Additional file 2


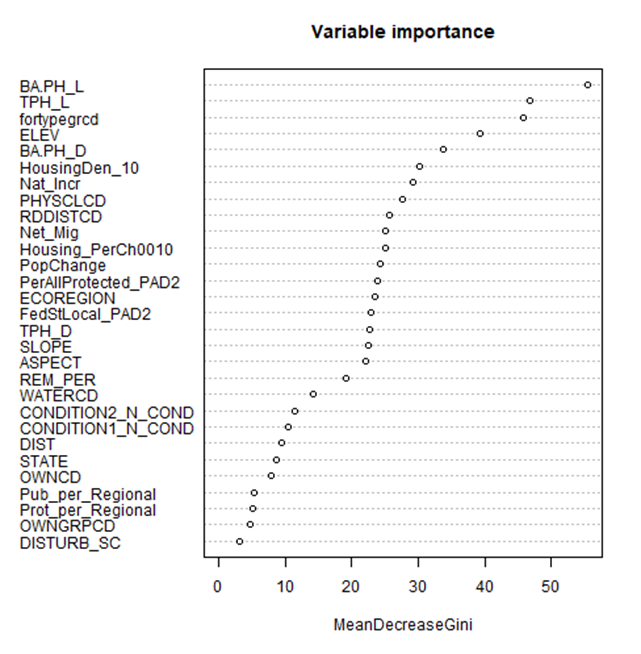


*Results:*

Forested plots that remained forested were well predicted by the random forest model (classification error was around 0.1%). However, the model failed to predict the plots that changed in land use (classification error over 99%) (Table 1). The overall out-of-bag (OOB) estimate of error rate was 3.43%.

Table 1: Confusion matrix predicting forest land use change for the random forest

| Class | No change | Change | Classification error |
| --- | --- | --- | --- |
| No change | 9712 | 11 | 0.0011 |
| Change | 334 | 3 | 0.9911 |

Legend: Random forest model was done with 500 trees and 5 variables tried at each split in six US states between 2000 - 2017

The five most important variables for the random forest classification model were live basal area, live trees per hectare, forest type, elevation, and dead basal area.

**Random forest for aboveground carbon stock changes**

For complete names of the variables, please see Additional file 3

**
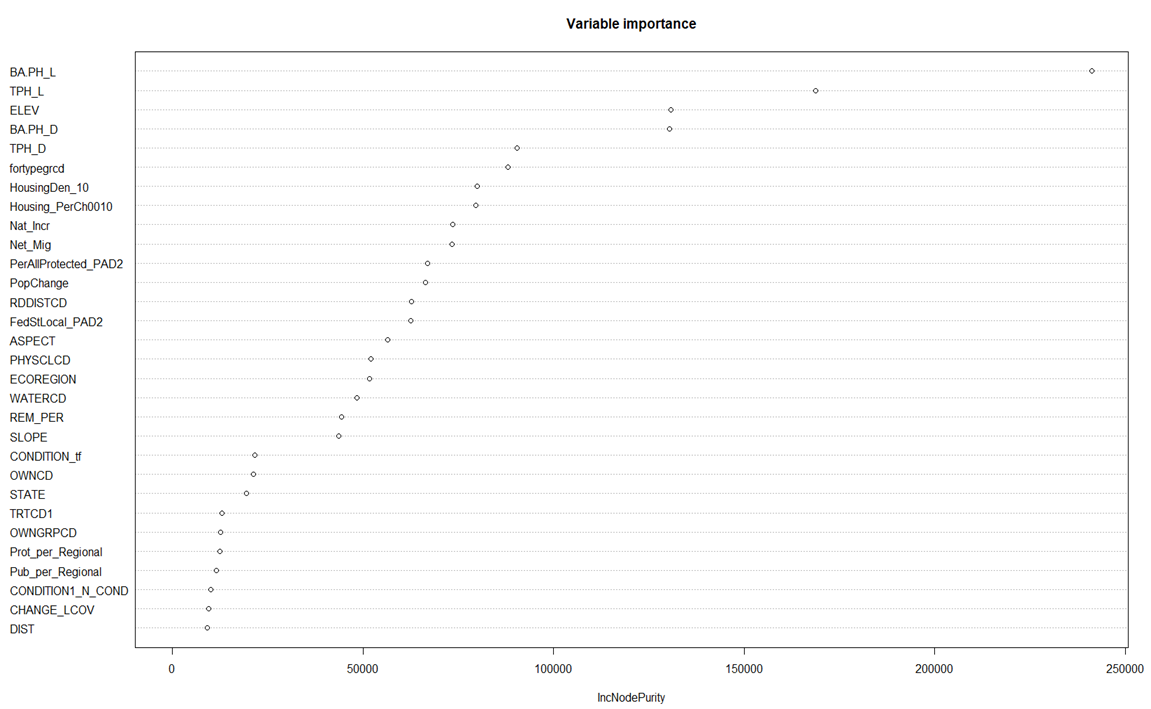
**

1. -Nwanganga F, Chapple M. Practical Machine Learning in R. Practical Machine Learning in R. 2020.

   -Ayyadevara VK. Pro Machine Learning Algorithms. Suresh CJ, Moodie M, Modi D, editors. Statistical Machine Learning. Springer; 2018. 379 p.

   -Pavlov YL. Random forests. The Neatherlands: Ridderprint bv; 2000. 122 p. [↑](#footnote-ref-1)
2. -Breiman L. Random forests. Mach Learn. 2001;28. [↑](#footnote-ref-2)
3. Wiener AL and M. Classification and Regression by randomForest. R News [Internet]. 2002;2(3):18–22. Available from: https://cran.r-project.org/doc/Rnews/ [↑](#footnote-ref-3)
