# Supplementary material for: Modeling land use change and forest carbon stock changes in temperate forests in the United States
Source: Carbon Balance Manag. 2021 Jul 3;16:20. doi: 10.1186/s13021-021-00183-6 (PMC8254905; doi:10.1186/s13021-021-00183-6)
Supplement: Supplementary file 2 — Additional file 2. Logistic regression model for land use change. [file 13021_2021_183_MOESM2_ESM.docx]

**Additional file 2**

**Logistic regression model for land use change**

*Variable list*

Groups

1. Forest attributes**

-Trees per hectare live (TPH_L)

-Trees per hectare dead (TPH_D)

-Basal area per hectare live (BA.PH_L)

-Basal area per hectare dead (BA.PH_D)

-Physiological classification code (PHYSCLCD)

-Ecoregion (ECOREGION): Derived from the ecological subsection code in FIA

-Ecological classification code (ECOSUBCD)

-Forest type code

-Forest type group code (fortypegrcd)

1. Plot attributes**

-Remeasurement period (REM_PER)

-Distance to the nearest road (RDDISTCD)

-Water code (WATERCD)

-Ownership code (OWNCD)

-Ownership group code (OWNGRPCD)

-County (COUNTYCD)

-State (STATECD)

1. Disturbances**

-Main disturbances (DIST): Derived from the disturbance code variable in FIA

-Disturbance simple/compound (DISTURB_SC): Derived from the disturbance code variable in FIA

1. Housing*

-Percentage of protected areas in a county (PerAllProtected_PAD2)

-Percentage of public land in a county (FedStLocal_PAD2)

-Percentiles of protected land by Census Region (Prot_per_Regional)

-Percentiles of public land (federal/state/local agencies) by Census Region (Pub_per_Regional)

-Change in the county population between 2000 and 2010 census (PopChange)

-Net migration between 2000 and 2010 census (Net_Mig)

-Natural increase between 2000 and 2010 census (Nat_Incr)

-Houses / km^2^ (HousingDen_10)

-Percent change in housing units between 2000 and 2010 (Housing_PerCh0010)

1. Forest condition**

-Number of forested conditions in a plot (CONDITION1_N_COND): Derived from the condition status code and condition ID variables from FIA

-Number of non-forested conditions in a plot (CONDITION2_N_COND): Derived from the condition status code and condition ID variables from FIA

1. Topography**

-Elevation (ELEV)

-Slope (SLOPE)

-Aspect (ASPECT)

**For metadata on these variables, refer to*: <https://doi.org/10.1016/j.jenvman.2018.03.053>

***For metadata on these variables, refer to*: <https://www.fia.fs.fed.us/library/database-documentation/#FIADB>

*Variable importance: Information Value*

Table 1: Variables used in the land use change model displaying their strength as predictor variables

| **Strong (IV< 0.02)** | **Medium (0.02 ≤ IV< 0.1)** | **Weak (0.1≤ IV < 0.3)** | **Not useful predictor (IV ≥ 0.3)** |
| --- | --- | --- | --- |
| - Ecological classification code (0.65) - Forest type (0.52) - Forest type group (0.39) - Ecoregion (0.35) - Remeasurement period (0.3) | - Basal area per hectare live (0.3) - Physiological classification code (0.2) - Basal area per hectare dead (0.2) - Trees per hectare dead (0.2) - State (0.18) - Trees per hectare live (0.13) - %Protected areas (0.1) | - Natural increment (0.1) - Ownership code (0.09) - Percentiles of protected land (0.09) - % of county land publicly administered (0.08) - House density (0.08) - Distance to the nearest road (0.07) - Number of non-forested conditions (0.07) - Percentiles of public land (0.07) - Slope (0.06) - Aspect (0.06) - Change in population (0.06) - Ownership group code (0.06) - Elevation (0.05) - Number of forested conditions (0.04) - Main disturbance (0.04) - Net migration (0.03) - Percent change in housing units (0.03) - Water code (0.02) | Disturbance: Simple/Compound (0.01) |

Legend: Variable strength was determined with the information value (IV) shown in brackets.

*Model equation and coefficients*

logit(Forest change p*_i_)~β_0_ +b_i_ +b_ij+_ β_1_ (Basal area live) + β_2_ (Basal area dead) + β_3_ (Remeasurement period) + α_Ownership code_ + β_4_ (%Protected areas) + β_5_(Natural increment) + β_6_(Non-forested conditions) + β_7_ (Forested conditions) + β_8_ (Aspect)*

*i = state*

*ij = state/forest type*

| **Variable** | ***Coefficient*** | ***SE*** | ***t*** | ***p*** | ***df*** |
| --- | --- | --- | --- | --- | --- |
| Intercept | 4.9989 | 1.0395 | 4.8088 | 0.0000 | 7848 |
| Trees per hectare live | 0.0019 | 0.0003 | 6.9126 | 0.0000 | 7848 |
| Live basal area | -0.1642 | 0.0187 | -8.7892 | 0.0000 | 7848 |
| Dead basal area | -0.1799 | 0.0513 | -3.5055 | 0.0005 | 7848 |
| Distance to road | -0.2007 | 0.0935 | -2.1472 | 0.0318 | 7848 |
| Disturbance_simple | -1.1523 | 0.5470 | -2.1068 | 0.0352 | 7848 |
| Disturbance_compound | -3.1088 | 1.9095 | -1.6281 | 0.1035 | 7848 |
| Percentage of protected areas in a county | -2.0876 | 0.7866 | -2.6539 | 0.0080 | 7848 |
| Number of forested conditions | 0.9456 | 0.3527 | 2.6814 | 0.0073 | 7848 |
| Condition at time 2_mix | -1.0596 | 0.3742 | -2.8317 | 0.0046 | 7848 |
| Condition at time 2_non-forest | -11.5062 | 1.9242 | -5.9796 | 0.0000 | 7848 |
| Change from forest to mix or non-forest (yes) | -3.3105 | 0.8244 | -4.0163 | 0.0001 | 7848 |
| Trees per hectare live: basal area live | 0.0000 | 0.0000 | -2.5394 | 0.0111 | 7848 |
