# Supplementary material for: Modeling land use change and forest carbon stock changes in temperate forests in the United States
Source: Carbon Balance Manag. 2021 Jul 3;16:20. doi: 10.1186/s13021-021-00183-6 (PMC8254905; doi:10.1186/s13021-021-00183-6)
Supplement: Supplementary file 3 — Additional file 3. Linear mixed-effects model for aboveground carbon changes. [file 13021_2021_183_MOESM3_ESM.docx]

**Additional file 3**

**Linear mixed-effects model for aboveground carbon changes**

*Variable list and metadata*

Groups

1. Forest attributes**

-Trees per hectare live (TPH_L)

-Trees per hectare dead (TPH_D)

-Basal area per hectare live (BA.PH_L)

-Basal area per hectare dead (BA.PH_D)

-Physiological classification code (PHYSCLCD)

-Ecoregion (ECOREGION): Derived from the ecological subsection code in FIA

-Ecological classification code (ECOSUBCD)

-Forest type code

-Forest type group code (fortypegrcd)

1. Plot attributes**

-Remeasurement period (REM_PER)

-Distance to the nearest road (RDDISTCD)

-Water code (WATERCD)

-Ownership code (OWNCD)

-Ownership group code (OWNGRPCD)

-County (COUNTYCD)

-State (STATECD)

1. Disturbances**

-Main disturbances (DIST): Derived from the disturbance code variable in FIA

-Disturbance simple/compound (DISTURB_SC): Derived from the disturbance code variable in FIA

1. Housing*

-Percentage of protected areas in a county (PerAllProtected_PAD2)

-Percentage of public land in a county (FedStLocal_PAD2)

-Percentiles of protected land by Census Region (Prot_per_Regional)

-Percentiles of public land (federal/state/local agencies) by Census Region (Pub_per_Regional)

-Change in the county population between 2000 and 2010 census (PopChange)

-Net migration between 2000 and 2010 census (Net_Mig)

-Natural increase between 2000 and 2010 census (Nat_Incr)

-Houses / km^2^ (HousingDen_10)

-Percent change in housing units between 2000 and 2010 (Housing_PerCh0010)

1. Forest condition**

-Number of forested conditions in a plot (CONDITION1_N_COND): Derived from the condition status code and condition ID variables from FIA

-Number of non-forested conditions in a plot (CONDITION2_N_COND): Derived from the condition status code and condition ID variables from FIA

-Condition at t_i_ : Derived from the condition status code from FIA

-Condition at t_f_ : Derived from the condition status code from FIA

-Change in land use (CHANGE_L): Derived from the variables Condition at t_i_ and Condition at t_f_ . Refers to all possible changes

-Change from forest (CHANGE_F):Derived from the variables Condition at t_i_ and Condition at t_f_ . Refers to changes from forest to mixed or non-forested conditions

1. Topography**

-Elevation (ELEV)

-Slope (SLOPE)

-Aspect (ASPECT)

**For metadata on these variables, refer to*: <https://doi.org/10.1016/j.jenvman.2018.03.053>

***For metadata on these variables, refer to*: <https://www.fia.fs.fed.us/library/database-documentation/#FIADB>

*Model equation and coefficients*

change_AG_carbon*_ij_*~ *β_0_ +b_i_ +b_ij_* +*β_1_*(Trees per hectare live) * *β_2_*(Basal area live)+ *β_3_*(Basal area dead) + *β_4_*(Distance to roads) + *α_Disturbance (simple/compound)_*+ *β_6_*(%Protected areas) + *β_7_*(Number of forested conditions) + *α_Condition at tf_* + *α_Change from forest_* +E*_ij_*

E*_ij_*~N (0,𝞼^2^)

*i = state*

*ij = state/forest type*

| **Variable** | ***Coefficient*** | ***SE*** | ***t*** | ***p*** | ***df*** |
| --- | --- | --- | --- | --- | --- |
| Intercept | 4.9989 | 1.0395354 | 4.8088 | 0.0000 | 7848 |
| Trees per hectare live | 0.0019 | 0.0 | 6.9126 | 0.0000 | 7848 |
| Live basal area | -0.1642 | 0.0 | -8.7892 | 0.0000 | 7848 |
| Dead basal area | -0.1799 | 0.1 | -3.5055 | 0.0005 | 7848 |
| Distance to road | -0.2007 | 0.1 | -2.1472 | 0.0318 | 7848 |
| Disturbance_simple | -1.1523 | 0.5 | -2.1068 | 0.0352 | 7848 |
| Disturbance_compound | -3.1088 | 1.9 | -1.6281 | 0.1035 | 7848 |
| Percentage of protected areas in a county | -2.0876 | 0.8 | -2.6539 | 0.0080 | 7848 |
| Number of forested conditions | 0.9456 | 0.4 | 2.6814 | 0.0073 | 7848 |
| Condition at time 2_mix | -1.0596 | 0.4 | -2.8317 | 0.0046 | 7848 |
| Condition at time 2_non-forest | -11.5062 | 1.9 | -5.9796 | 0.0000 | 7848 |
| Change from forest to mix or non-forest (yes) | -3.3105 | 0.8 | -4.0163 | 0.0001 | 7848 |
| Trees per hectare live: basal area live | 0.0000 | 0.0 | -2.5394 | 0.0111 | 7848 |
