# Supplementary material for: Modeling land use change and forest carbon stock changes in temperate forests in the United States
Source: Carbon Balance Manag. 2021 Jul 3;16:20. doi: 10.1186/s13021-021-00183-6 (PMC8254905; doi:10.1186/s13021-021-00183-6)
Supplement: Supplementary file 4 — Additional file 4. Wall-to-wall maps of the probability of forest areas to convert to non-forest separated by state. [file 13021_2021_183_MOESM4_ESM.pdf]

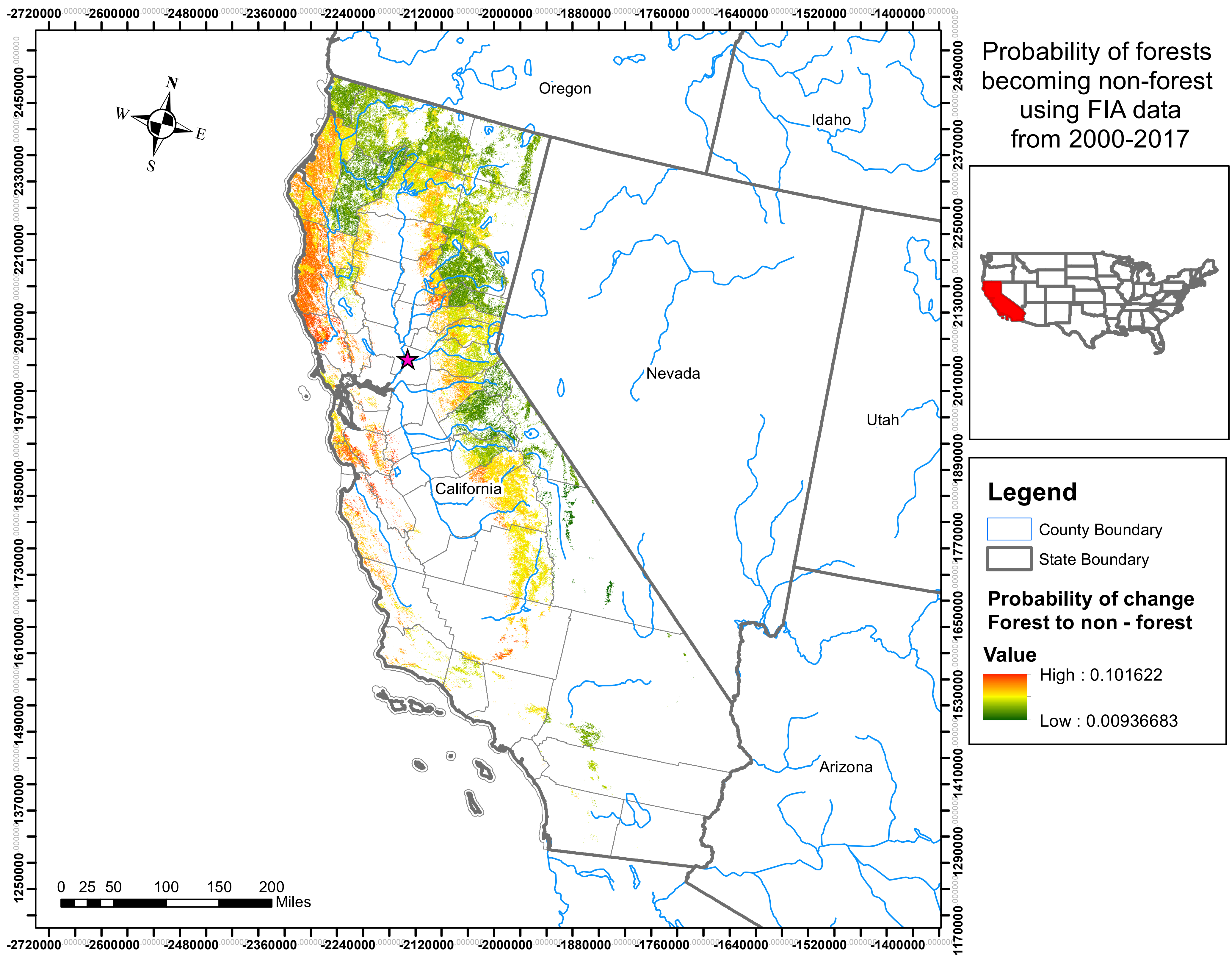

Probability of forests becoming non-forest using FIA data from 2000-2017
Legend
County Boundary
State Boundary
Probability of change Forest to non - forest
Value
High : 0.101622
Low : 0.00936683
Oregon
Idaho
Nevada
Utah
California
Arizona
N
S
E
W
0 25 50 100 150 200
Miles
-2720000
-2600000
-2480000
-2360000
-2240000
-2120000
-2000000
-1880000
-1760000
-1640000
-1520000
-1400000
2490000
2450000
2370000
2330000
2250000
2210000
2130000
2090000
2010000
1970000
1890000
1850000
1770000
1730000
1650000
1610000
1530000
1490000
1410000
1370000
1290000
1250000
1170000

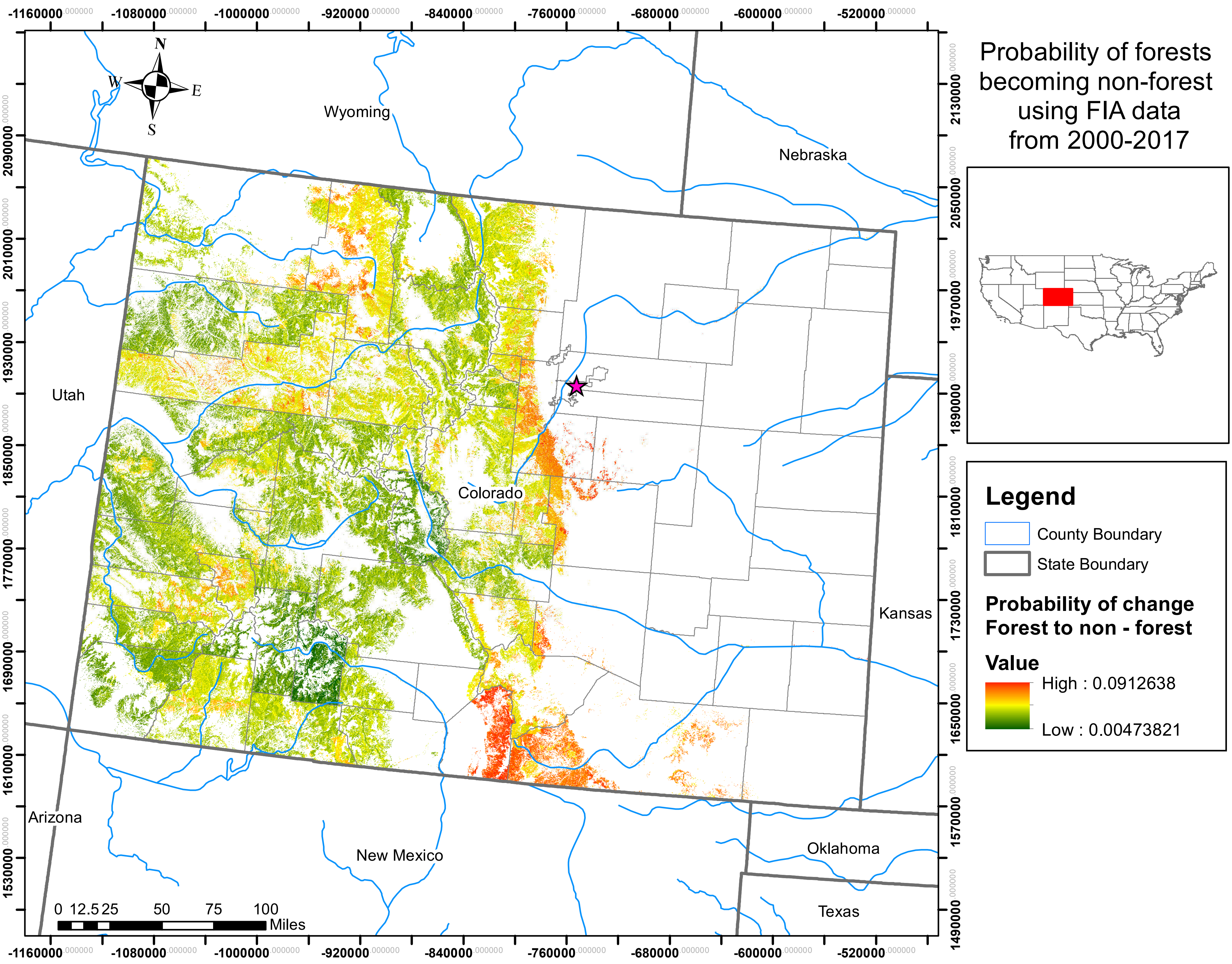
Probability of forests becoming non-forest using FIA data from 2000-2017
Legend
County Boundary
State Boundary
Probability of change Forest to non - forest
Value
High : 0.0912638
Low : 0.00473821
Wyoming
Nebraska
Utah
Colorado
Kansas
Arizona
New Mexico
Oklahoma
Texas
N
S
E
W
0 12.5 25 50 75 100 Miles
-1160000
-1080000
-1000000
-920000
-840000
-760000
-680000
-600000
-520000
2130000
2090000
2050000
2010000
1970000
1930000
1890000
1850000
1810000
1770000
1730000
1690000
1650000
1610000
1570000
1530000
1490000

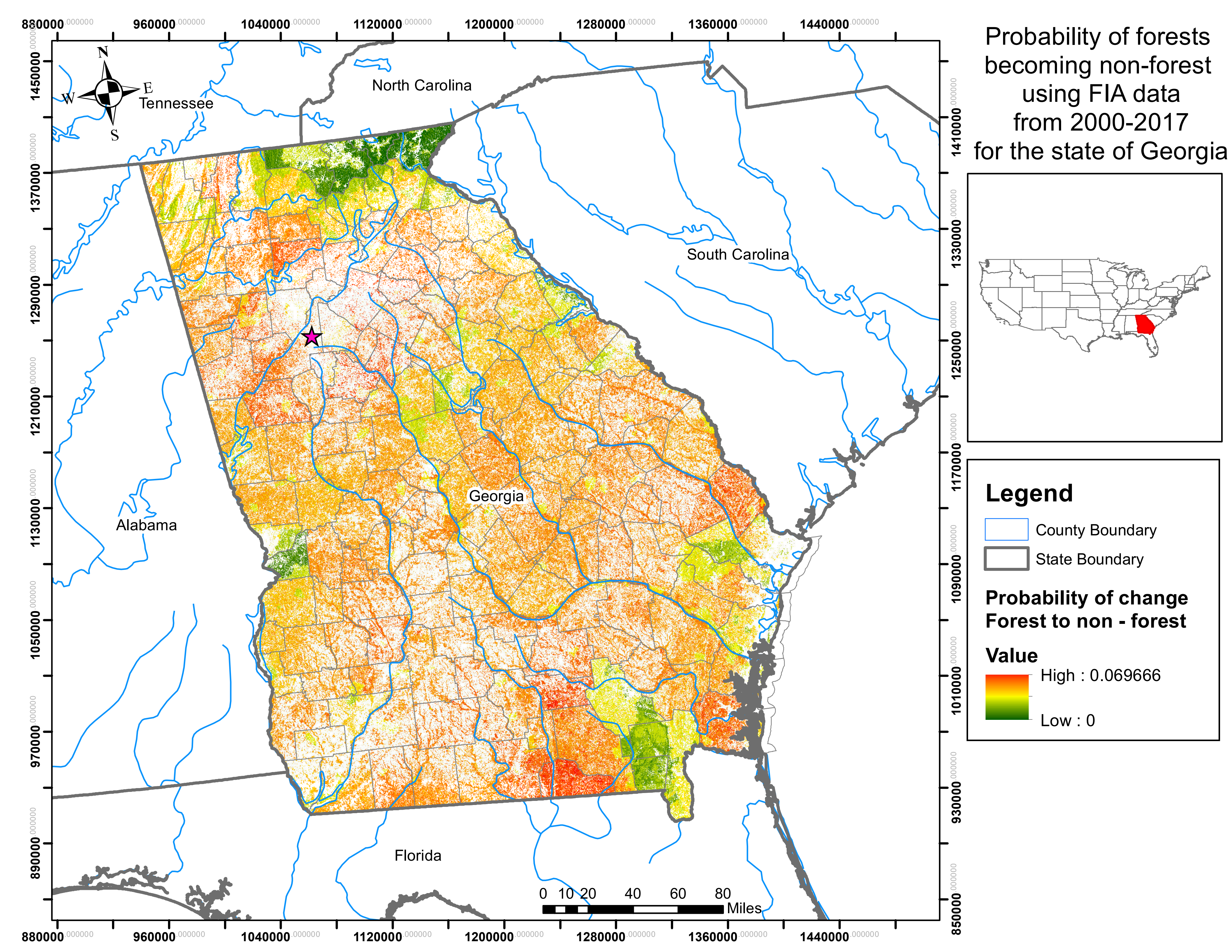
Probability of forests becoming non-forest using FIA data from 2000-2017 for the state of Georgia
Legend
County Boundary
State Boundary
Probability of change Forest to non - forest
Value
High : 0.069666
Low : 0
North Carolina
Tennessee
South Carolina
Georgia
Alabama
Florida
N
E
S
W
0 10 20 40 60 80 Miles
880000
960000
1040000
1120000
1200000
1280000
1360000
1440000
1450000
1370000
1290000
1210000
1130000
1050000
970000
890000
1410000
1330000
1250000
1170000
1090000
1010000
930000
850000

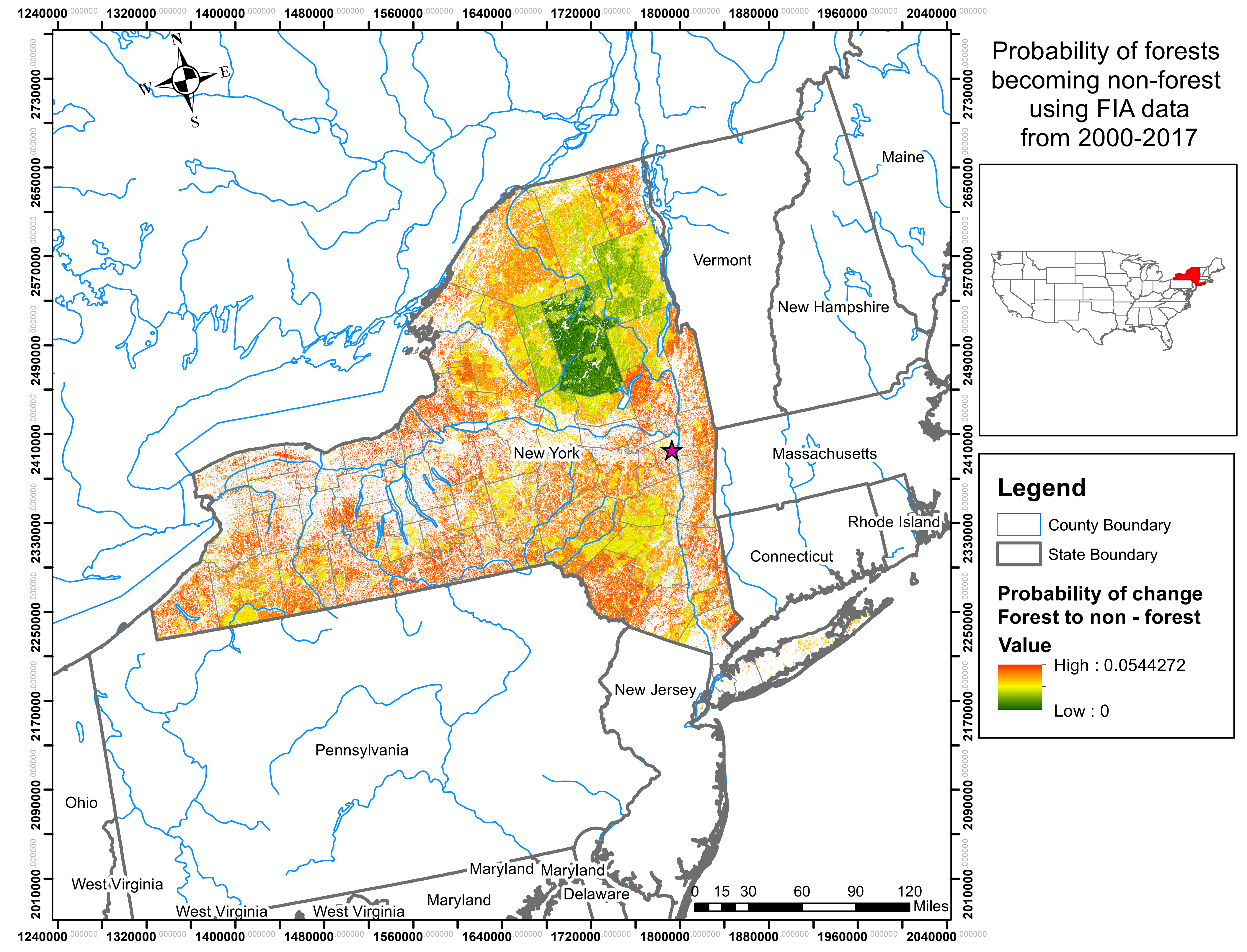
Probability of forests becoming non-forest using FIA data from 2000-2017
Legend
County Boundary
State Boundary
Probability of change Forest to non - forest
Value
High : 0.0544272
Low : 0
N
E
S
W
Vermont
Maine
New Hampshire
Massachusetts
Rhode Island
Connecticut
New York
New Jersey
Pennsylvania
Ohio
West Virginia
West Virginia
West Virginia
Maryland
Maryland
Maryland
Delaware
0 15 30 60 90 120
Miles
1240000 1320000 1400000 1480000 1560000 1640000 1720000 1800000 1880000 1960000 2040000
2730000 2650000 2570000 2490000 2410000 2330000 2250000 2170000 2090000 2010000

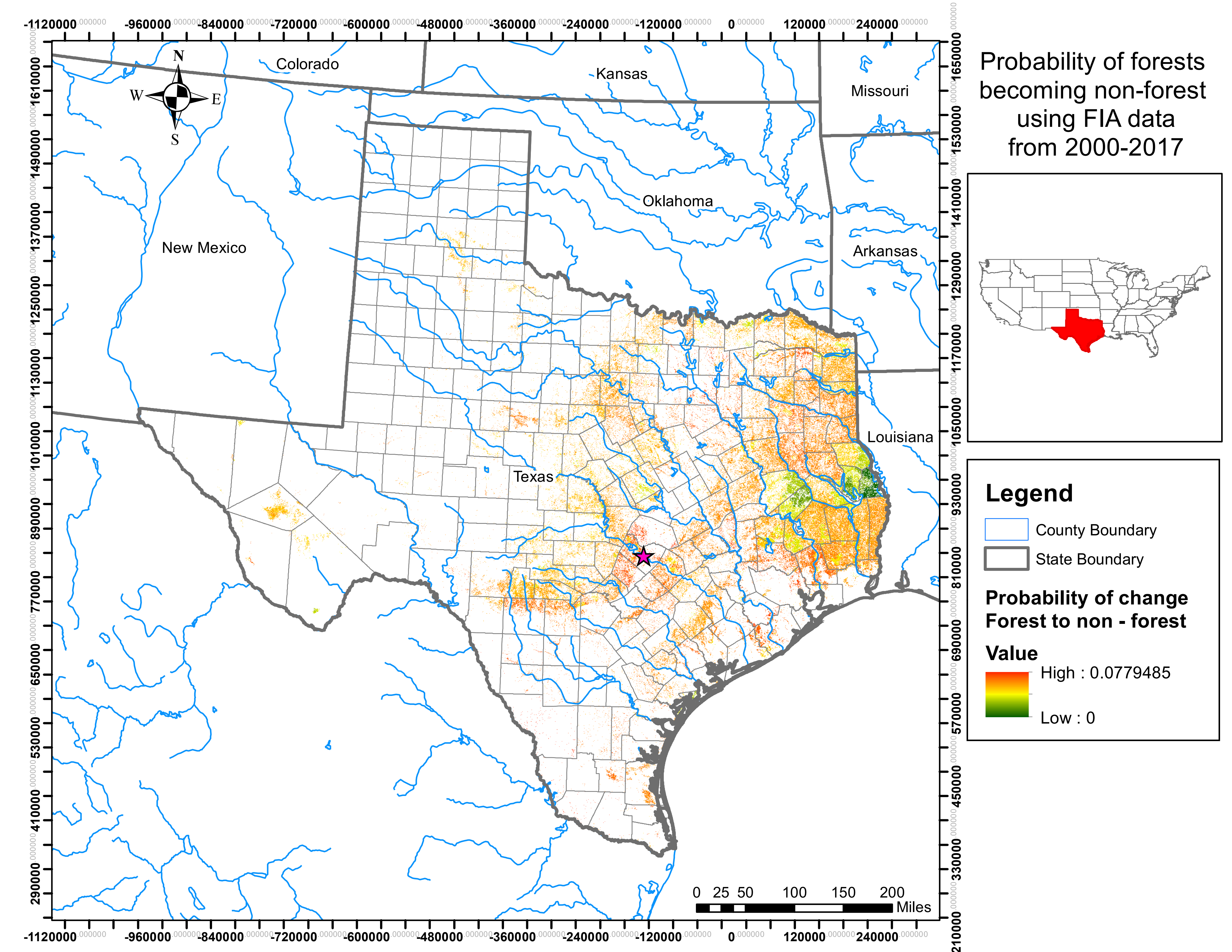
Probability of forests becoming non-forest using FIA data from 2000-2017
Colorado
Kansas
Missouri
Oklahoma
New Mexico
Arkansas
Texas
Louisiana
N
S
E
W
Legend
County Boundary
State Boundary
Probability of change Forest to non - forest
Value
High : 0.0779485
Low : 0
0 25 50 100 150 200
Miles
-1120000
-960000
-840000
-720000
-600000
-480000
-360000
-240000
-120000
0
120000
240000
1650000
1610000
1530000
1490000
1410000
1370000
1290000
1250000
1170000
1130000
1050000
1010000
930000
890000
810000
770000
690000
650000
570000
530000
450000
410000
330000
290000
210000

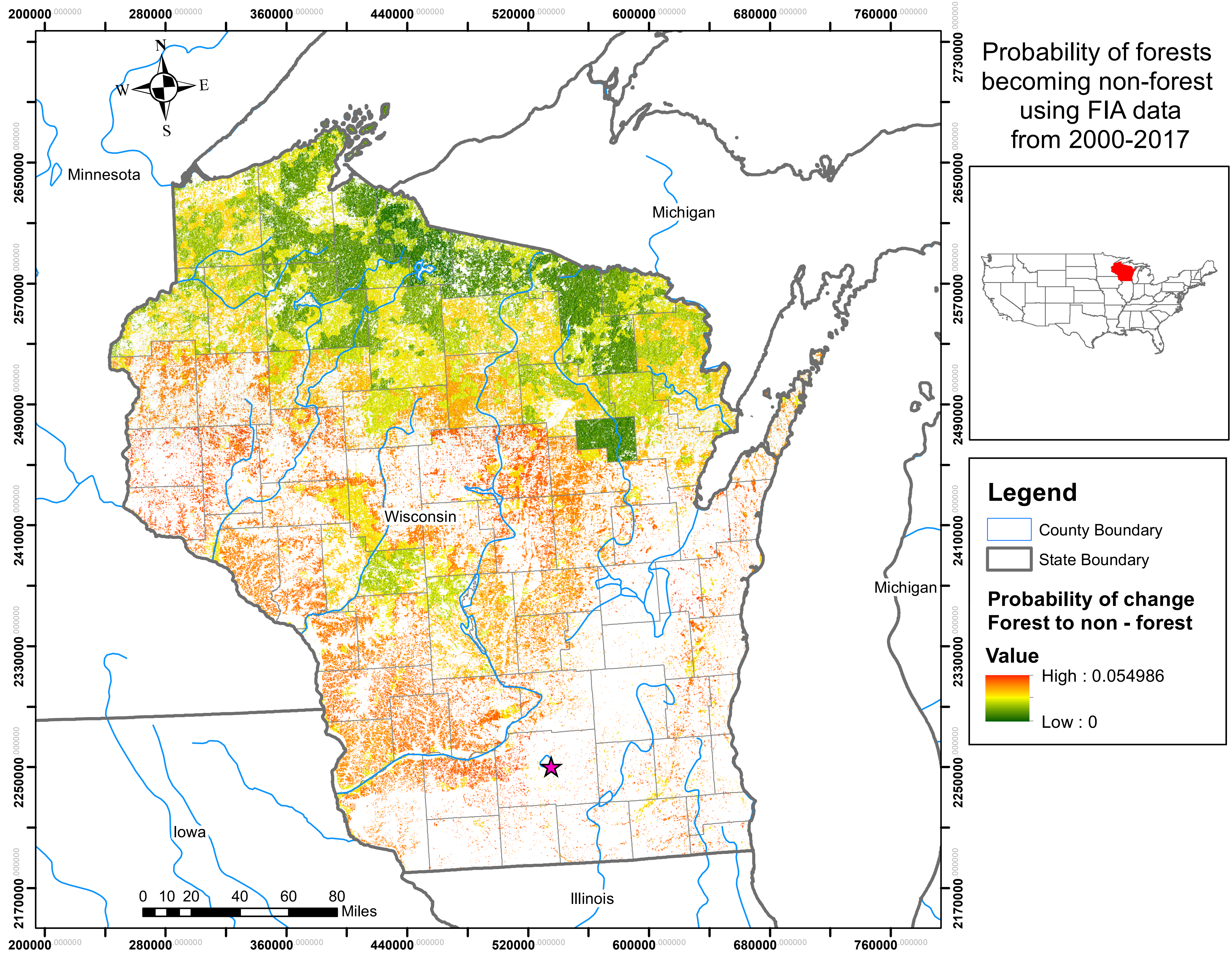

Probability of forests becoming non-forest using FIA data from 2000-2017
Legend
County Boundary
State Boundary
Probability of change Forest to non - forest
Value
High : 0.054986
Low : 0
Minnesota
Michigan
Wisconsin
Michigan
Iowa
Illinois
N
E
S
W
0 10 20 40 60 80
Miles
200000
280000
360000
440000
520000
600000
680000
760000
2730000
2650000
2570000
2490000
2410000
2330000
2250000
2170000
